# TRESK K^+^ Channel Activity Regulates Trigeminal Nociception and Headache

**DOI:** 10.1523/ENEURO.0236-19.2019

**Published:** 2019-07-26

**Authors:** Zhaohua Guo, Chang-Shen Qiu, Xinghua Jiang, Jintao Zhang, Fengxian Li, Qin Liu, Ajay Dhaka, Yu-Qing Cao

**Affiliations:** 1 Washington University Pain Center, Washington University School of Medicine, St. Louis, Missouri 63110; 2 Department of Anesthesiology, Washington University School of Medicine, St. Louis, Missouri 63110; 3 Center for the Study of Itch, Washington University School of Medicine, St. Louis, Missouri 63110; 4 Department of Biological Structure, Neurobiology and Behavior Graduate Program, University of Washington, Seattle, Washington 98195

**Keywords:** headache, intrinsic excitability, primary afferent neuron, TRESK, trigeminal ganglion, trigeminal pain

## Abstract

Although TWIK-related spinal cord K^+^ (TRESK) channel is expressed in all primary afferent neurons in trigeminal ganglia (TG) and dorsal root ganglia (DRG), whether TRESK activity regulates trigeminal pain processing is still not established. Dominant-negative TRESK mutations are associated with migraine but not with other types of pain in humans, suggesting that genetic TRESK dysfunction preferentially affects the generation of trigeminal pain, especially headache. Using TRESK global knock-out mice as a model system, we found that loss of TRESK in all TG neurons selectively increased the intrinsic excitability of small-diameter nociceptors, especially those that do not bind to isolectin B4 (IB4^−^). Similarly, loss of TRESK resulted in hyper-excitation of the small IB4^−^ dural afferent neurons but not those that bind to IB4 (IB4^+^). Compared with wild-type littermates, both male and female TRESK knock-out mice exhibited more robust trigeminal nociceptive behaviors, including headache-related behaviors, whereas their body and visceral pain responses were normal. Interestingly, neither the total persistent outward current nor the intrinsic excitability was altered in adult TRESK knock-out DRG neurons, which may explain why genetic TRESK dysfunction is not associated with body and/or visceral pain in humans. We reveal for the first time that, among all primary afferent neurons, TG nociceptors are the most vulnerable to the genetic loss of TRESK. Our findings indicate that endogenous TRESK activity regulates trigeminal nociception, likely through controlling the intrinsic excitability of TG nociceptors. Importantly, we provide evidence that genetic loss of TRESK significantly increases the likelihood of developing headache.

## Significance Statement

TRESK K^+^ channel is expressed in all primary afferent neurons in trigeminal ganglia (TG) and dorsal root ganglia (DRG), but dominant-negative TRESK mutations are only associated with migraine but not with other types of pain in humans. In TRESK global knock-out mice, we found that ubiquitous loss of TRESK selectively increased the intrinsic excitability of small-diameter TG nociceptors without affecting DRG neuronal excitability. Compared with wild-type littermates, TRESK knock-out mice exhibited more robust trigeminal pain, especially headache-related behaviors, whereas their body and visceral pain responses were normal. This recapitulates the clinical manifestations of human TRESK mutations. Our results indicate that endogenous TRESK activity regulates trigeminal nociception, and genetic loss of TRESK significantly increases the likelihood of developing headache.

## Introduction

The TWIK-related spinal cord K^+^ (TRESK) channel belongs to the two-pore domain K^+^ (K_2P_) family of background K^+^ channels and is encoded by the KCNK18 gene (Sano et al., 2003; Kang et al., 2004; Enyedi and Czirják, 2015). TRESK is abundantly expressed in primary afferent neurons (PANs) in trigeminal ganglia (TG) and dorsal root ganglia (DRG) but at a negligible level in other tissues, suggesting that its main physiologic function is to control somatosensation via regulating PAN excitability (Czirják et al., 2004; Dobler et al., 2007; Yoo et al., 2009; Lafrenière et al., 2010; Kollert et al., 2015). Indeed, previous studies indicate that TRESK is one of the major background K^+^ channels in DRG neurons (Kang and Kim, 2006; Marsh et al., 2012). It is well established that TRESK activity regulates the excitability of DRG neurons and controls the generation of body pain under both normal and disease conditions. Both nerve injury and tissue inflammation reduce the level of TRESK mRNA in rat DRG (Tulleuda et al., 2011; Marsh et al., 2012). Inhibition of TRESK and other K_2P_ channels by sanshool robustly increases the firing of DRG mechanoreceptors (Bautista et al., 2008; Lennertz et al., 2010). Reducing DRG TRESK activity elicits nocifensive behavior and increases mechanical sensitivity on the hindpaw (Tulleuda et al., 2011; Zhou et al., 2016). Overexpression of TRESK in rat DRG neurons inhibits neuropeptide release and attenuates neuropathic pain (Zhou et al., 2012, 2013).

Much less is known about whether and how endogenous TRESK activity regulates trigeminal pain processing, especially the trigeminovascular pathway subserving headache. Dominant-negative TRESK mutations are associated with migraine but not with other types of pain in humans (Lafrenière et al., 2010), suggesting that genetic TRESK dysfunction differentially affects the generation of trigeminal pain and body pain. However, a recent study suggests that TRESK mutations increase TG neuronal excitability not through reducing TRESK current per se, but though inhibiting other K_2P_ channels TREK1/2 by the product of alternative translation initiation from the mutant KCNK18 allele (Royal et al., 2019). This calls into question the role of endogenous TRESK activity in controlling headache and other trigeminal pain. Many outstanding questions remain unanswered. Does endogenous TRESK activity regulate the intrinsic excitability of TG neurons, especially dural afferent neurons, the PANs in the trigeminovascular pathway? Does TRESK dysfunction enhance headache and other trigeminal nociception at the behavioral level? Can DRG neurons tolerate genetic TRESK dysfunction and maintain their intrinsic excitability, thereby exhibiting normal transduction of noxious stimuli that evoke body pain in adulthood?

In this study, we used the TRESK global knock-out (KO) mice as a model system to address these questions. We found that loss of TRESK in all TG neurons selectively increased the intrinsic excitability of small-diameter nociceptors, especially those that do not bind to isolectin B4 (IB4^−^). Notably, some small IB4^−^ TG neurons from KO mice exhibited spontaneous action potentials (sAPs) at resting membrane potential (*V*_rest_), whereas sAP was not present in any of the wild-type (WT) TG neurons. Loss of TRESK also resulted in hyper-excitation of the small IB4^−^ dural afferent neurons but not those that bind to IB4 (IB4^+^). In DRG neurons, neither the total persistent outward current nor the intrinsic excitability was affected by the genetic loss of TRESK. Together, we reveal for the first time that, among all primary afferent neurons, TG nociceptors are the most vulnerable to the genetic loss of TRESK. Compared with WT littermates, both male and female TRESK KO mice exhibited more robust trigeminal nociceptive behaviors, especially headache-related behaviors, but displayed normal responses in body pain and visceral pain models. This recapitulates the clinical presentations of human TRESK mutations. Collectively, our findings indicate that endogenous TRESK activity regulates trigeminal nociception, likely through controlling the intrinsic excitability and suppressing sAPs in TG nociceptors. Importantly, our results indicate that genetic loss of TRESK significantly increases the likelihood of developing headache.

## Materials and Methods

### Mice

All procedures were conducted in strict accordance with the recommendations in Society for Neuroscience's Policies on the Use of Animals and humans in Neuroscience Research and the guidelines of the Institutional Animal Care and Use Committee at Washington University in St. Louis. To avoid social isolation stress, all mice were group housed (2–5 per cage, same sex) in the animal facility of Washington University in St. Louis on a 12 h light/dark cycle with constant temperature (23–24°C), humidity (45–50%), and food and water *ad libitum*. All mice were maintained on C57BL/6J background (backcrossed for at least 10 generations). Adult male and female littermates (8–20 weeks old) were used in the behavioral tests and immunohistochemistry (IHC) experiments. Young adult mice (5–7 weeks old) were used in the electrophysiology experiments. This is based on the preliminary results showing that the total and lamotrigine-sensitive outward currents as well as the intrinsic excitability of PANs remained stable between 5 and 10 weeks old WT mice, but the abundance of medium-sized neurons was lower in cultures from older mice.

WT and TRESK KO mice were generated by crossing heterozygous (HET) breeders (Kcnk18^tm1(KOMP)Vlcg^, KOMP repository). We also crossed HET TRESK breeders with mice that express enhanced green fluorescent protein (EGFP) from one TRPM8 locus (TRPM8^EGFP^; Dhaka et al., 2008) and mice that contain a transgenic allele that expresses EGFP under the control of the genomic sequences that regulate the expression of endogenous vesicular glutamate transporter 3 (VGLUT3^EGFP^; Seal et al., 2009). The double-HET breeders were then crossed with TRESK heterozygotes to generate WT/KO_TRPM8^EGFP^ and WT/KO_VGLUT3^EGFP^ mice, respectively. Genotypes were determined by PCR of tail DNA as described previously (Valenzuela et al., 2003; Dhaka et al., 2008; Seal et al., 2009). For TRESK allele genotyping, the WT allele was amplified with primers TUF (5′-GAGGAGAACCCTGAGTTGAAGAAG-3′) and TUR (5′-GCACCTCCGAGGCAGTAAC-3′), producing a 103 bp fragment. The targeted allele was amplified with primers NeoInF (5′-TTCGGCTATGACTGGGCACAACAG-3′) and NeoInR (5′-TACTTTCTCGGCAGGAGCAAGGTG-3′), producing a 282 bp fragment. The PCR conditions were 96°C for 30 s, 50°C for 30 s, and 72°C for 30 s for 40 cycles.

### Primary culture of mouse TG and DRG neurons

TG and lumbar DRG (L3–L5) tissues were collected from 5 to 7 weeks old WT and TRESK KO mice of either sex and were treated with 2.5 mg/ml collagenase intravenously followed by 2.5 mg/ml trypsin at 37°C for 15 min, respectively. Cells were dissociated by triturating with fire-polished glass pipettes and were centrifuged through a 15% BSA gradient to remove debris and non-neuronal cells. Neurons were resuspended in MEM-based culture medium containing 5% fetal bovine serum, 25 ng/ml NGF, 10 ng/ml GDNF, and were seeded on Matrigel-coated coverslips. Electrophysiology recordings were performed in neurons 2–4 d *in vitro* (DIV). Each set of experiment contains neurons from at least three batches of culture.

### Electrophysiology

Whole-cell patch-clamp recordings were performed at room temperature (RT) with a MultiClamp 700B amplifier (Molecular Devices). The recording chamber was perfused with Tyrode’s solution (0.5 ml/min) containing the following (in mm): 130 NaCl, 2 KCl, 2 CaCl_2_, 2 MgCl_2_, 25 HEPES, 30 glucose, pH 7.3 with NaOH, and 310 mOsm/kgH_2_O. The pipette solution contained the following (in mm): 130 K-gluconate, 7 KCl, 2 NaCl, 0.4 EGTA, 1 MgCl_2_, 4 ATP-Mg, 0.3 GTP-Na, 10 HEPES, 10 Tris-phosphocreatine, 10 U/ml creatine phosphokinase, pH 7.3 with KOH, and 290 mOsm/kgH_2_O. Recording pipettes had <4.5 MΩ resistance. pClamp 10 (Molecular Devices) was used to acquire and analyze data. Cell capacitance and series resistance were constantly monitored throughout the recording. Data were analyzed with the Clampfit (Molecular Devices) and Origin (OriginLab) software.

Neurons were recorded between 2 and 4 DIV. Longer culture time did not alter the number or the thickness of the processes but significantly increased the length and the branches of individual processes. The processes of cultured neurons would contribute to the space-clamp error. We did not find significant differences in the size of persistent outward current or neuronal excitability between early (2 DIV) and late (4 DIV) recordings within individual experimental groups. Thus the space clamp issue was not exacerbated by the prolonged culture time.

#### Voltage-clamp experiments

Series resistance (<15 MΩ, average 12 ± 1 MΩ) was compensated by 80%. Current traces were not leak subtracted. Signals were filtered at 2 kHz and digitized at 20 kHz. Total persistent outward K^+^ current and current through TRESK channels in neurons were recorded as described previously (Guo and Cao, 2014). Briefly, the extracellular solution contained 1 µm tetrodotoxin (TTX) to block TTX-sensitive Na^+^ current. To minimize other transient voltage-gaged Na^+^, K^+^, and Ca^2+^ currents, we held neurons at −60 mV and depolarized them to −25 mV for 150 ms, and then ramped the potential to −135 mV at 0.37 mV/ms every 10 s. The current at the end of the −25 mV depolarizing step was measured as the total persistent outward current. To further dissect K^+^ currents through TRESK channels, we bath-applied 30 µm lamotrigine (Sigma-Aldrich) while evoking whole-cell currents using this pulse protocol (Guo and Cao, 2014).

#### Current-clamp experiments

Series resistance (<15 MΩ) was not compensated. Signals were filtered at 10 kHz and digitized at 100 kHz. After whole cell access was established, membrane capacitance was determined with amplifier circuitry. The amplifier was then switched to current-clamp mode to measure *V*_rest_. Input resistant (*R*_in_) was calculated by measuring the membrane potential change in response to a 20 pA hyperpolarizing current injection from *V*_rest_. Neurons were excluded from analysis if the *V*_rest_ was >−40 mV or *R*_in_ was <200 MΩ.

To test neuronal excitability, neurons at *V*_rest_ were injected with 1 s depolarizing currents in 5 or 25 pA incremental steps. The rheobase was defined as the minimum amount of current required to elicit at least one action potential (AP). The first AP elicited using this paradigm was used to measure AP threshold (the membrane potential at which *dV*/*dt* exceeds 10 V/s), amplitude, and half-width. The afterhyperpolarization (AHP) amplitude was measured from the single AP elicited by injecting a 1 ms depolarizing current in 200 pA incremental steps from the *V*_rest_.

Differential interference contrast images of neurons were captured before the recording to calculate soma diameters from cross-sectional areas off-line. At the end of electrophysiological recording, neurons were incubated with 3 µg/ml Fluorescein isothiocyanate (FITC)-conjugated IB4 (Sigma-Aldrich) for 5 min. The FITC fluorescence on soma membrane was detected after 10 min perfusion with Tyrode’s solution to wash off unbound IB4. The recording pipette remained attached to the neurons during IB4 staining and washing. The *V*_rest_, *R*_in_, capacitance, series resistance, and leak currents were not significantly altered during the process.

### Tissue preparation, IHC, and image analysis

Adult WT and TRESK KO mice were euthanized with intraperitoneal injection of barbiturate (200 mg/kg) and were transcardially perfused with 0.1 m PBS, pH 7.2, followed by 4% formaldehyde in 0.1 m PB, pH 7.2, for fixation. TGs and DRGs were dissected out, postfixed for 4 h, and protected overnight at 4°C in 0.1 m PB with 30% sucrose. The entire TG or DRG was then frozen in optimal cutting temperature compound, sectioned at 15 µm in the transverse plane using a cryostat, collected on Superfrost Plus glass slides in sequence and stored at −20°C.

One in every three TG or DRG sections was processed for each IHC experiment. The sections were dried at RT, washed three times in 0.01 m PBS, and incubated in blocking buffer consisting 0.01 m PBS, 10% NGS, and 0.3% triton X-100 for 1 h at RT. Sections were then incubated overnight with primary antibodies diluted in blocking buffer in a humidity chamber at 4°C. After six washes (5 min each) in washing buffer containing 0.01 m PBS with 1% NGS and 0.3% triton and 1 h incubation in blocking buffer, sections were incubated with secondary antibodies (1:1000 dilution in blocking buffer) at RT for 1 h, washed six times with washing buffer and rinsed three times in 0.01 m PBS. Sections were then coverslipped using Fluoromount-G Slide Mounting Medium (Electron Microscopy), sealed with nail polish, and stored at 4°C.

Sections were costained with the rabbit anti-βIII tubulin antibody (Covance; 1:1000) and the chicken anti-TRESK antibody (1:1000; generated in-house as described by Liu et al., 2013) recognizing the first extracellular loop of the mouse TRESK subunit. AlexaFluor 568-conjugated goat anti-rabbit and AlexaFluor 488-conjugated goat anti-chicken secondary antibodies (Invitrogen) were used at 1:1000 dilutions. To count total TG neurons, images of the entire TG sections containing βIII tubulin-IR were captured using an Olympus NanoZoomer Whole-Slide Imaging System. All neurons on individual sections were counted and the total number of βIII tubulin-positive neurons was multiplied by 3 to obtain the total number of TG neurons for each mouse (Golden et al., 2010). Representative images were adjusted for contrast and brightness using the same parameter within individual experiments. No other manipulations were made to the images.

To quantify macrophages in adult WT and KO TG, sections were stained with a rabbit antibody recognizing ionized calcium binding adaptor molecule 1 (Iba1, Wako; 1:1000) and the AlexaFluor 568-conjugated goat anti-rabbit secondary antibody (Invitrogen; 1:1000). Non-overlapping images of the TG sections were randomly captured through a 20× objective on a Nikon TE2000S inverted epifluorescence microscope equipped with a CoolSnapHQ2 camera (Photometrics). The number and the cross-sectional area of Iba1-positive (Iba1^+^) macrophages were measured using the SimplePCI software (Hamamatsu).

### Retrograde labeling of TG neurons innervating the dura

We used FluoroGold (FG; 2% in saline; Fluorochrome) to label the dural afferent neurons in 6-weeks old WT and TRESK KO mice as described in a previous study (Huang et al., 2012). Briefly, mice were anesthetized with 3–4% isoflurane in an induction chamber until losing the righting reflex and were mounted on a Stoelting stereotaxic apparatus. Anesthesia was maintained by 1.5–2% isoflurane through a nose cone. Body temperature was maintained by placing mice on a 37°C circulating water-warming pad. The eyes were covered by a small amount of eye drops to prevent the corneas from drying. A longitudinal skin incision was made to expose the cranium. The muscle and periosteal sheath were removed. Lidocaine hydrochloride jelly (2%) was applied on the skin and the skull for 5 min before the incision and the muscle/sheath removal to prevent the activation and/or sensitization of the primary afferent fibers. A craniectomy (∼2.5 mm diameter) was made with a surgical blade in the area overlying the superior sagittal sinus between the bregma and lambda, leaving the underlying dura exposed but intact. To prevent spreading of the tracer to other peripheral sites, a sterile polypropylene ring was sealed to the skull surrounding the exposed dura using a mixture of dental cement powder (Stoelting, 51459) and superglue adhesive. After waiting 5–10 min for the mixture to solidify, we applied 20 μl of FG onto the exposed dura. Subsequently, a sterile polypropylene cap was secured over the ring with the dental cement/superglue mix to cover the exposed dura. The skin incision was closed with 5-0 suture. After recovery from anesthesia, mice were housed individually in the animal facility to allow the transportation of FG to the somata of dural afferent neurons in TG. TG tissues were collected 7 d after dural application of FG for primary culture and electrophysiology recording. To measure the soma size distribution of dural afferent neurons, TG tissues were collected after the transcardial perfusion fixation.

### Behavioral tests

WT and TRESK KO mice were generated by crossing heterozygous breeders on C57BL/6J background. Adult male and female littermates (8–20 weeks old) were used in the behavioral tests. The experimenters were blinded to the genotype of mice.

#### General motor function tests

*Open field test.* Mice were habituated in the testing room for 1 h in their home cages and then tested one at a time. Each mouse was placed in the center of the illuminated, sound-attenuated VersaMax Open Field box (42 ×42 cm; AccuScan Instruments) for 1 h while the experimenter left the room. Horizontal movement and center entries (14 × 14 cm) were recorded and analyzed by the VersaMax software.

*Rotarod test.* Mice were habituated in the testing room for 1 h in their home cages and then underwent 5 training sessions on the Rotarod (Ugo Basile, model 7650) at 4 rpm for 5 min. Mice that stayed on the rod for at least 2 min/session were tested on the accelerating rod (4–40 rpm over 5.5 min). The latency to fall from the rod was averaged over five trials in individual mice.

*Responses to noxious stimuli on the hindpaw.* Each mouse was acclimatized in the test apparatus for 1–2 h before the application of stimuli.

*Mechanical stimuli.* A series of calibrated von Frey filaments was used to apply mechanical stimuli to the plantar surface of the hindpaw. We used the up–down paradigm to determine the 50% withdrawal threshold (Chaplan et al., 1994).

*Thermal and cold stimuli.* We recorded the latency to lick the hindpaw and/or to jump on a 55°C hot plate. The cutoff time was 20 s. We also measured the paw withdrawal latencies to radiant heat stimuli applied to the plantar surface of the hindpaw (Hargreaves et al., 1988). The cold plantar assay was used to compare the response latencies of WT and TRESK KO mice to cold stimuli on the plantar surface of the hindpaw (Brenner et al., 2012).

*Chemical stimuli.* Mice were injected with 0.3 µg of capsaicin (in 10 µl saline with 1% DMSO) in the plantar surface of one hindpaw. We quantified the time spent licking the treated paw within 5 min after the injection.

*Visceral pain test.* We counted the number of abdominal stretches that occurred within 30 min after intraperitoneal injection of 5.0 ml/kg 0.6% acetic acid, a stimulus that produces visceral pain with inflammation (Cao et al., 1998).

#### Responses to facial noxious stimuli

*Eye-blink responses to thermal stimuli.* Mice were well habituated to the test apparatus and extensively handled by the experimenter for a week before testing. On the test day, the experimenter gently held the mouse on the palm and delivered the thermal stimulus by blowing air to one eye at 0.5 L/min for 10 s (Huang et al., 2018). The number of eye blinks was recorded by a video camera and quantified off-line. The distance between the air outlet (6.35 mm diameter) and the eye was 2–3 mm. The air was heated to various temperatures (20–55°C) by passing through plastic tubing submerged in heated water bath. The air temperature was measured by a thermal probe at the outlet. Mice were tested every 3 d. Each eye was tested twice per day, first with RT air and 1–2 h later with air at a higher temperature. Results from left and right eyes were averaged for individual mice.

*Cheek acetone test.* We measured the acute nocifensive responses to acetone-evoked evaporative cooling on mouse cheek (Constandil et al., 2012; Trevisan et al., 2016). Mice were habituated individually in clear Plexiglas boxes (11 × 11 × 15 cm) situated in front of three angled mirrors for at least 1 h for 3 consecutive days and were extensively handled by the experimenter. The day before testing, both cheeks were shaved (6.5 × 12 mm) under brief anesthesia (2% isoflurane in 100% oxygen). On the test day, mice were habituated for 1 h before the assay. We applied acetone (15 µl) to the shaved cheeks and immediately returned mice to the boxes. Time spent wiping the treated area was recorded by a video camera and quantified off-line. For individual mice, acetone was applied alternatingly to both cheeks at >10 min interval, and the duration of behavior was averaged from four applications.

*Cheek capsaicin injection.* Mice were acclimated and shaved as in the facial acetone test. On the test day, we habituated mice in the clean boxes for 1 h and then injected 1 µg of capsaicin (in 20 µl saline with 1% DMSO) or vehicle intradermally in one cheek (Shimada and LaMotte, 2008; Akiyama et al., 2010). Mice were immediately returned to the boxes and recorded by video cameras for 40 min. Total numbers of forepaw wiping of the injected cheek were quantified off-line.

*Operant behavioral responses to thermal stimuli.* Responses to facial thermal stimuli were tested in the Orofacial Pain Assessment Device (OPAD; Stoelting), which uses a reward-conflict paradigm that allows a mouse to choose between receiving a reward (sweetened condensed milk diluted 1:2 with water) and escaping an aversive stimulus (50°C thermal stimuli on the cheeks), thereby controlling the amount of pain it feels. First, mice underwent four training sessions in 2 weeks. The day before each session, mice were food deprived for 16 h and both cheeks were shaved. The next day, individual mice were trained in the OPAD for 20 min to learn to voluntarily press the cheeks onto the Peltier bars set at 33˚C to drink sweetened milk (Anderson et al., 2013). Mice that consistently licked >600 times/per session were used to test facial thermal responses. Trained mice underwent two test sessions 3 d apart. During each test session, the thermode temperature varied every 3 min as follows: 33–50– 33–50–33°C. The number of licks and the number of thermode contacts at each temperature were recorded and averaged in individual mice.

*Withdrawal responses to facial mechanical stimuli.* Mice were well habituated to the test room and extensively handled by the experimenter for at least 2 weeks before testing. The hair on the forehead (above and between 2 eyes) was shaved the day before testing. On the test day, the experimenter gently held the mouse on the palm with minimal restraint and applied the calibrated von Frey filament perpendicularly to the shaved skin, causing the filament to bend for 5 s. A positive response was determined by the following criteria as previously described: mouse vigorously stroked its face with the forepaw, head withdrawal from the stimulus, or head shaking (Elliott et al., 2012). We used the up–down paradigm to determine the 50% withdrawal threshold (Chaplan et al., 1994).

*Mouse model of headache.* Craniectomy was performed on 9- to 15-week-old WT and TRESK KO mice as described in a previous study (Huang et al., 2016) and in the section of retrogradely labeling of dural afferent neurons above. We applied 20 µl of sterile saline to the dura and let mice recover from surgery for 7 d. Mice were housed individually in the animal facility and were transported to the testing room for acclimation and handling every day. On the test day, mice in their home cages were habituated in the testing room for 1 h and were briefly anesthetized with isoflurane. The polypropylene cap was removed without disturbing the skin suture and 20 µl of vehicle (saline with 2% DMSO) or IScap solution was applied onto the exposed dura. The composition of IScap solution was detailed at the end of the section. Subsequently, a new sterile polypropylene cap was secured over the ring with bone wax to cover the dura. After recovery from anesthesia, mice were returned to the home cages placed in front of a three-way mirror to ensure that the head-directed behavior be recorded at all body positions. Home cage behaviors were recorded 0.5–2.5 h after the dural application. Mice were recorded one at a time in the absence of the experimenter and other mice. Digital video files were quantified off-line by experimenters blinded to the genotypes of mice or the treatments that mice received. The entire 2 h video was watched and scored. The total number and the duration of forepaw wiping and hindpaw scratching within the mouse V_1_ dermatome (including the scalp and periorbital area) were quantified.

IScap was not washed away from the dura once applied, because we intended to simulate the prolonged activation/sensitization of dural afferent neurons during migraine headache. In a pilot experiment, we found that there was no increase in V1-directed nocifensive behavior if we removed the dura in WT mice and directly applied IScap to the cortex, indicating that IScap-induced behavior likely resulted from the activation of meningeal nociceptors, not the leaking of IScap below the dura (data not shown). In another pilot experiment, we applied the dye fast green (MW 808 Da) to the dura surface for 2 h and examined the spread of fast green. There was no detectable fast green signal in the superior sagittal sinus or the cortex (data not shown). The average diameter of the FG-stained area on the dura was 8.6 ± 0.5 mm (Huang et al., 2016). Neither WT nor TRESK KO mice receiving IScap displayed increased grooming behaviors in V2/V3 areas (data not shown). We did not quantify grooming behavior on the body. We did not observe nocifensive behaviors such as paw gnawing in any mice receiving IScap or vehicle.

The IScap solution contained the following (in mm): 0.5 capsaicin, 1 bradykinin, 1 histamine, 1 serotonin, and 0.1 prostaglandin E2 (PGE_2_) in saline with 2% DMSO. All chemicals were purchased from Sigma-Aldrich, dissolved in H_2_O (bradykinin, histamine, and serotonin) or DMSO (capsaicin and PGE_2_) at 100× concentrations and stored at −80°C in aliquots. IScap solution was freshly prepared from the stock solutions on the test day.

### Experimental design and statistical analysis

WT, HET, and KO littermates were used and were tested in parallel in individual experiments. For electrophysiology experiments, data from each group contained neurons from at least three independent cultures. Each culture included at least two mice in each group. For behavioral experiments, power analysis is conducted to estimate sample size with >80% power to reach a significance level of 0.05. The experimenters were blinded to the genotype of mice. For IHC experiments, sample sizes were estimated based on our prior experience.

All data are reported as mean ± SEM. The Shapiro–Wilk test was used to check data normality. Statistical significance between experimental groups was assessed by Fisher’s exact test, χ^2^ test, two-tailed *t* test, ANOVA [one-way or two-way, with or without repeated-measures (RM)] with the *post hoc* Bonferroni test where appropriate, using Origin and Statistica (from OriginLab and StatSoft, respectively). The non-parametric Kruskal-Wallis ANOVA on ranks with Tukey’s *post hoc* pairwise comparison was used to analyze the differences in the withdrawal threshold to mechanical stimuli. Differences with *p* < 0.05 were considered statistically significant. The statistical analysis for individual experiments was described in figure legends. All data generated or analyzed in this study will be deposited to the Digital Research Materials Repository of Washington University in St. Louis and are available on reasonable request.

## Results

### TRESK protein is ubiquitously expressed in mouse PANs

**Figure 1.**
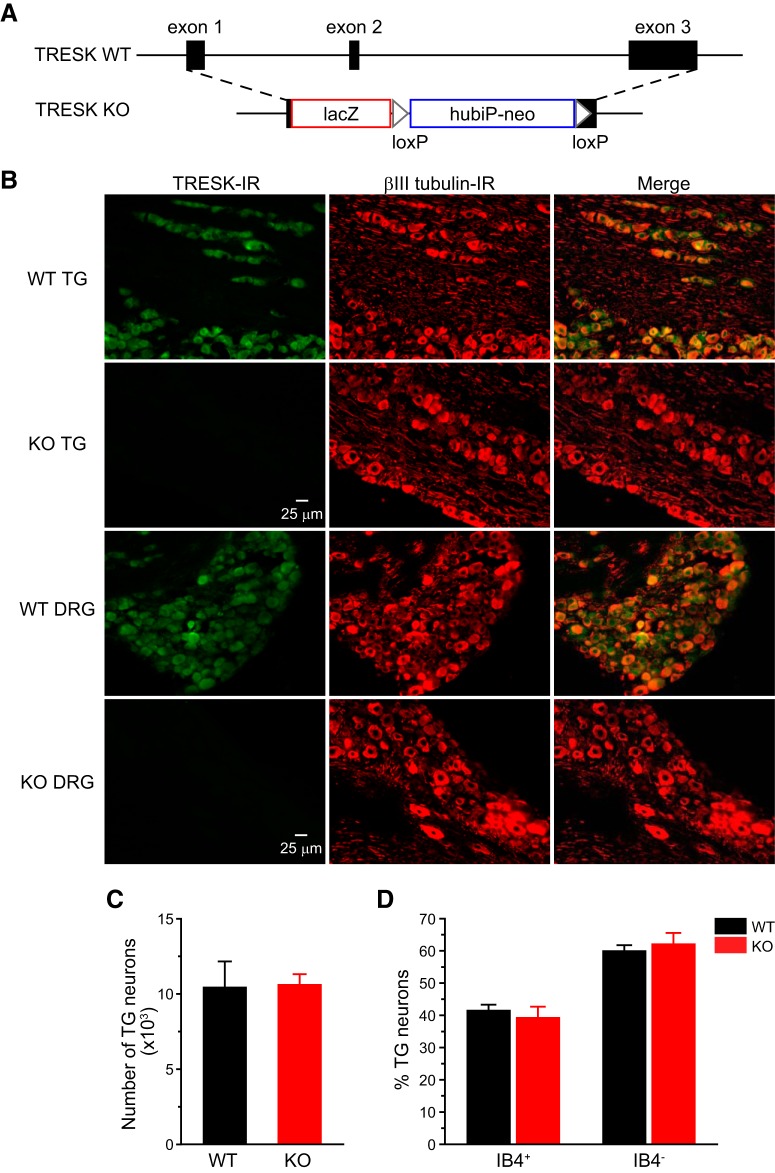
Functional TRESK channels are present in all TG and DRG neurons. ***A***, Schematics of WT mouse TRESK/Kcnk18 allele and the targeted allele. After homologous recombination, the lacZ-loxP-hubiP-neo-loxP cassette replaces most of the TRESK coding region. ***B***, Representative images of TG and DRG sections from adult WT and TRESK KO mice double stained with antibodies against TRESK and βIII tubulin. βIII tubulin-IR is present in all neurons. TRESK-IR is present in almost all neurons in the WT sections. There is no TRESK-IR in the KO sections. ***C***, Total number of TG neurons in WT and TRESK KO mice (*n* = 3 mice in each group). ***D***, The percentage of IB4^+^ and IB4^–^ neurons in WT and KO TG culture (1010 WT and 940 KO TG neurons from 10 separate primary cultures were counted).

TRESK KO mice (Fig. 1*A*) were grossly normal. Progenies from HET crossing had the expected Mendelian frequency. Both male and female KO mice gained weight normally and were fertile, with normal litter size and maternal behavior. First, we examined the distribution of TRESK protein in TG and DRG tissues. We stained TG and DRG sections from adult WT and KO mice with an antibody that recognizes mouse TRESK protein (Liu et al., 2013; Guo and Cao, 2014). TRESK-IR was completely absent in tissues from KO mice (Fig. 1*B*), validating the specificity of the antibody. In TG and DRG sections from WT mice, we used an βIII tubulin antibody to label all neurons (Golden et al., 2010) and found that TRESK-IR overlapped with βIII tubulin signal in almost all neurons (Fig. 1*B*), indicating that TRESK protein is ubiquitously expressed in mouse PANs but not in non-neuronal cells. This is consistent with the *in situ* hybridization and immunohistochemistry results in previous studies (Dobler et al., 2007; Yoo et al., 2009; Lafrenière et al., 2010; Guo and Cao, 2014; Kollert et al., 2015).

Next, we asked whether loss of TRESK affects the gross development of TG in mice. The total number of TG neurons containing βIII tubulin-IR did not differ between adult WT and TRESK KO mice (Fig. 1*C*), suggesting that TRESK is not required for the survival of PANs. The abundance of TG neurons that binds to IB4 was also comparable between cultured TG neurons from adult WT and KO mice (Fig. 1*D*). We then focused on investigating how loss of TRESK affects the functions of PANs and the behavioral responses to noxious stimuli.

### Loss of TRESK in TG neurons selectively increases the excitability of small-diameter nociceptors

**Figure 2.**
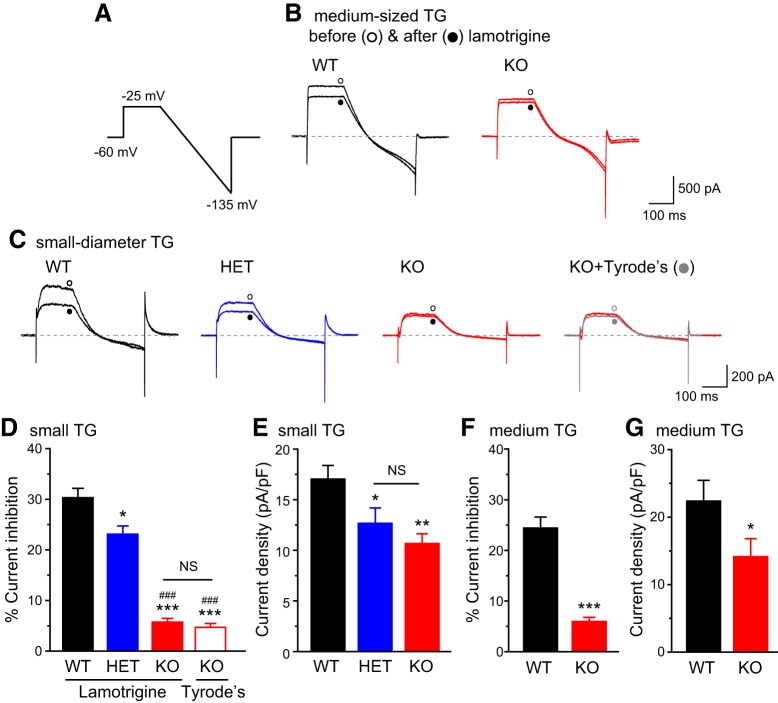
**Total persistent outward currents and lamotrigine-sensitive K^+^ currents in TG neurons from WT, HET, and TRESK KO mice. *A***, The voltage protocol used to record persistent outward currents and to minimize transient voltage-gated K^+^, Na^+^, and Ca^2+^ currents. ***B***, Representative current traces from medium-sized (25–40 µm diameter) WT and KO TG neurons before and after the application of 30 µm lamotrigine. ***C***, Representative current traces from small-diameter (<25 µm) WT, HET, and KO TG neurons before and after the application of 30 µm lamotrigine (or Tyrode’s solution), respectively. ***D***, ***E***, The percentage of lamotrigine-sensitive persistent K^+^ currents (***D***) and the total persistent outward current density (***E***) in small-diameter TG neurons (*n* = 14–20 neurons in each group, all measured at the end of the step to −25 mV). *p < 0.05, **p < 0.01, ***p < 0.001; one-way ANOVA with *post hoc* Bonferroni test compared with the WT group; ###*p* < 0.001; compared with the HET group. NS, No statistically significant difference. ***F***, ***G***, The percentage of lamotrigine-sensitive persistent K^+^ currents (***F***) and the total persistent K^+^ current density (***G***) in medium-sized TG neurons from WT and KO mice (*n* = 15 and 21 neurons, respectively). *p < 0.05, ***p < 0.001; two-tailed *t* test.

In previous studies, we used the sensitivity to lamotrigine to dissect currents through TRESK channels (Liu et al., 2013; Guo and Cao, 2014; Guo et al., 2014). Bath application of 30 µm lamotrigine blocked 20–30% of persistent outward currents in every adult WT TG neurons that we recorded, regardless of soma size (Fig. 2*A*–*D*,*F*). The same concentration of lamotrigine did not significantly reduce the outward currents in TG neurons from TRESK KO mice (Fig. 2*B*–*D*,*F*, no difference between lamotrigine- and Tyrode’s-treated KO neurons), indicating that the majority of lamotrigine-sensitive current in TG neurons are mediated through TRESK channels under our recording conditions. The size of total persistent outward current was also significantly reduced in KO TG neurons (Fig. 2*E*,*G*). TG neurons from HET mice showed intermediate levels of lamotrigine-sensitive TRESK current and total persistent outward current (Fig. 2*C*–*E*).

**Figure 3.**
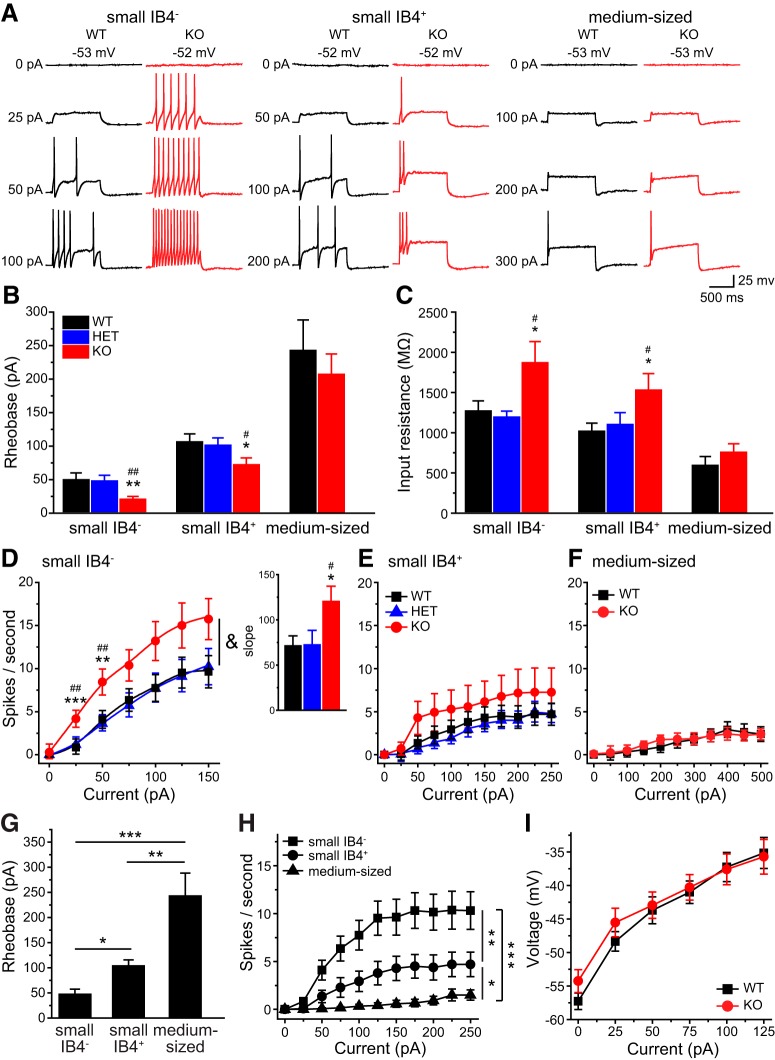
**Ubiquitous loss of TRESK currents preferentially increases the excitability of small IB4^−^ TG neurons. *A***, Representative traces of APs generated by incremental depolarizing current injections in TG neurons from WT and TRESK KO mice. The values of *V*_rest_ and the current amplitude are indicated. ***B***, ***C***, Mean rheobase (***B***; the minimum amount of current required to elicit at least 1 AP) and *R*_in_ (***C***) of subpopulations of TG neurons from TRESK WT, HET, and KO mice (*n* = 14–28 neurons in each group; for details of the intrinsic properties of TG neurons, see Table 1). *p < 0.05, **p < 0.01; one-way ANOVA with *post hoc* Bonferroni test compared with the corresponding WT group; #*p* < 0.05, ##*p* < 0.01; compared with the corresponding HET group. ***D***–***F,*** Input/output plots of the spike frequency in response to incremental depolarizing current injections in small IB4^−^ (***D***), small IB4^+^ (***E***) and medium-sized (***F***) TG neurons from TRESK WT, HET, and KO mice (same neurons as in ***B***). ^&^*p* < 0.05; two-way RM ANOVA; TRESK KO neurons versus WT and HET groups. **p < 0.01, ***p < 0.001; one-way ANOVA with *post hoc* Bonferroni test compared with the corresponding WT group; ##*p* < 0.01; compared with the corresponding HET group. Inset, The slope (spikes/sec/nA) of the input/output relationships between 25 and 125 pA current injections. *p < 0.05, #*p* < 0.05; one-way ANOVA with *post hoc* Bonferroni test compared with the WT and HET groups, respectively. ***G***, Mean rheobase of small IB4^−^, small IB4^+^, and medium-sized TG neurons from WT mice (same neurons as in ***B***, WT groups). *p < 0.05, **p < 0.01, ***p < 0.001; one-way ANOVA with *post hoc* Bonferroni test. ***H***, Input/output plots of the spike frequency in response to incremental depolarizing current injections in subpopulations of WT TG neurons (same WT neurons as in ***B*** WT groups). *p < 0.05, **p < 0.01, ***p < 0.001; two-way RM ANOVA. ***I***, The plot of steady-state membrane potential versus injected current in medium-sized TG neurons from WT and KO mice (same neurons as in ***B***, medium-sized groups).

We proceeded to investigate how loss of TRESK affects TG neuronal excitability. The mean *V*_rest_ was comparable between adult WT, HET and TRESK KO TG neurons (Table 1), consistent with previous studies suggesting that endogenous TRESK activity does not regulate *V*_rest_ in PANs (Dobler et al., 2007; Liu et al., 2013; Guo et al., 2014). To test how ubiquitous loss of TRESK current affects the excitability of TG neurons, we divided TG neurons into three subpopulations and used current-clamp recording to compare the passive and active electrophysiological properties of WT, HET, and TRESK KO neurons within each group. Neurons were first sorted by soma diameter into small- (<25 µm) and medium-sized (25–40 µm) groups. The majority of small TG neurons are nociceptors (Harper and Lawson, 1985a,b), with a small subset representing the C-low threshold mechanoreceptors (C-LTMRs) that transmit innocuous touch sensation (Lawson et al., 1997; Seal et al., 2009). Most of the medium-sized TG neurons are low-threshold mechanoreceptors with myelinated Aβ fibers (Goldstein et al., 1991; Perry et al., 1991; Bae et al., 2015). Small TG neurons were further divided into IB4^+^ and IB4^−^ groups, based on their ability to bind to fluorescently labeled IB4. It is well established that the small IB4^+^ and IB4^−^ PANs exhibit distinct neurochemical, anatomic, and electrophysiological properties and encode different pain modalities (Snider and McMahon, 1998; Stucky and Lewin, 1999; Fang et al., 2006; Choi et al., 2007; Cavanaugh et al., 2009; Scherrer et al., 2009). Indeed, both the rheobase value and the spike frequency were significantly different between small IB4^−^, small IB4^+^, and medium-sized TG neurons from WT mice (Fig. 3*A*,*G*,*H*).

**Table 1.** Intrinsic properties of TG neurons from adult WT, HET, and TRESK KO mice

	Diameter, μm	Capacitance, pF	*R*_in_, MΩ	*V*_rest_, mV	Rheobase, pA	AP threshold, mV	AP amplitude, mV	AP half-width, ms	AHP amplitude, mV	Cell number
Small IB4-negative neurons										
WT	18.3 ± 0.6	18.0 ± 1.6	1249 ± 126	−50.8 ± 1.1	47 ± 10	−14.7 ± 1.9	104.5 ± 3.0	5.3 ± 0.6	19.6 ± 0.9	28
HET	18.6 ± 0.5	16.8 ± 1.8	1172 ± 74	−52.8 ± 1.1	45 ± 9	−16.4 ± 1.4	105.6 ± 1.5	3.5 ± 0.4	17.3 ± 1.0	16
KO^a^	18.1 ± 0.6	17.3 ± 1.7	1849 ± 262*	−50.1 ± 1.3	18 ± 4**	−18.5 ± 1.7	98.9 ± 2.7	4.7 ± 0.5	17.5 ± 1.8	17
Small IB4-positive neurons										
WT	19.2 ± 0.5	20.0 ± 1.2	997 ± 99	−53.3 ± 1.6	103 ± 12	−10.7 ± 2.6	110.2 ± 4.1	5.4 ± 0.8	19.8 ± 0.9	14
HET	22.5 ± 0.7	20.6 ± 1.6	1080 ± 149	−54.7 ± 2.0	99 ± 11	16.1 ± 1.9	112.2 ± 2.8	5.7 ± 0.7	17.2 ± 1.0	17
KO	20.1 ± 1.0	22.8 ± 2.9	1507 ± 205*	−53.4 ± 1.4	70 ± 10*	13.6 ± 1.4	107.8 ± 3.8	6.9 ± 0.7	18.2 ± 2.2	14
Medium-sized neurons										
WT	30.8 ± 0.2	40.6 ± 2.5	593 ± 110	−57.8 ± 0.9	243 ± 46	15.0 ± 2.5	119.5 ± 3.0	4.9 ± 0.5	16.6 ± 0.2	16
KO	29.3 ± 0.1	40.2 ± 2.7	754 ± 108	−56.3 ± 1.1	207 ± 30	14.7 ± 1.8	113.5 ± 3.5	5.0 ± 0.7	17.6 ± 0.3	21

*p < 0.05, **p < 0.01; one-way ANOVA with *post hoc* Bonferroni test, compared with the corresponding WT and HET groups.

a Neurons that exhibit spontaneous APs at *V*_rest_ are not included.

First, we investigated whether loss of TRESK affects the excitability of small IB4^−^ TG neurons, as the majority of these neurons express CGRP, the neuropeptide that plays an important role in migraine pathophysiology (Price and Flores, 2007; Tao et al., 2012; Edvinsson and Goadsby, 2019). Compared with WT neurons, small IB4^−^ TG neurons from KO mice exhibited a significantly higher *R*_in_ and consequently a >50% reduction of rheobase for AP generation (Fig. 3*A*–*C*). The values of AP threshold, amplitude, half-width, and AHP amplitude were not affected by the loss of TRESK (Table 1), suggesting that TRESK current plays a negligible role in shaping the AP waveforms.

In both WT and KO small IB4^−^ TG neurons, the number of APs initially increased almost linearly in response to incremental depolarizing current injection and plateaued on further depolarization (Fig. 3*D*). The slope of the input-output curve between 25 and 125 pA current injections was significantly steeper in the KO group (Fig. 3*D*, inset), and the same amount of depolarizing current evoked more APs in KO neurons than in WT (Fig. 3*D*). We conclude that the endogenous TRESK currents control the onset and the frequency of APs in small IB4^−^ TG neurons.

Next, we compared the excitability of small IB4^+^ TG neurons from WT and KO mice. Loss of TRESK also resulted in higher *R*_in_ and lower rheobase in this TG subgroup (Fig. 3*A*–*C*). In WT TG culture, the input/output curve of IB4^+^ neurons was much flatter than that of small IB4^−^ neurons (Fig. 3*H*). Loss of TRESK did not significantly increase the spike frequency in small IB4^+^ TG neurons (Fig. 3*E*), indicating that the endogenous TRESK activity regulates AP initiation but not AP frequency in this TG subpopulation.

Among the three subtypes of WT TG neurons, the medium-sized neurons had the lowest *R*_in_, highest rheobase and the majority of them generated a single spike in response to both threshold and suprathreshold current injections (Fig. 3*B*,*C*,*G*,*H*; Ratté et al., 2014). Surprisingly, loss of TRESK did not alter *R*_in_, rheobase or spike frequency in medium-sized TG neurons at all (Fig. 3*A*–*C*,*F*), despite the significant reduction of total persistent outward current at −25 mV (Fig. 2*F*). We found that injection of depolarizing currents induced similar membrane potential changes between –60 mV and −35 mV in WT and KO medium-sized TG neurons (Fig. 3*I*), suggesting that there is little endogenous TRESK activity around *V*_rest_ to oppose the membrane depolarization in WT medium-sized TG neurons. Indeed, using data from medium-sized TG neurons in Figure 2, we found that the mean density of lamotrigine-sensitive currents in WT neurons at –40 mV was only 1.70 ± 0.26 pA/pF. At this voltage, the density of total persistent outward current in WT and KO medium-sized neurons were not statistically different (10.8 ± 1.5 pA/pF and 8.0 ± 1.6 pA/pF, respectively, *p* = 0.23, two-tailed *t* test).

**Figure 4.**
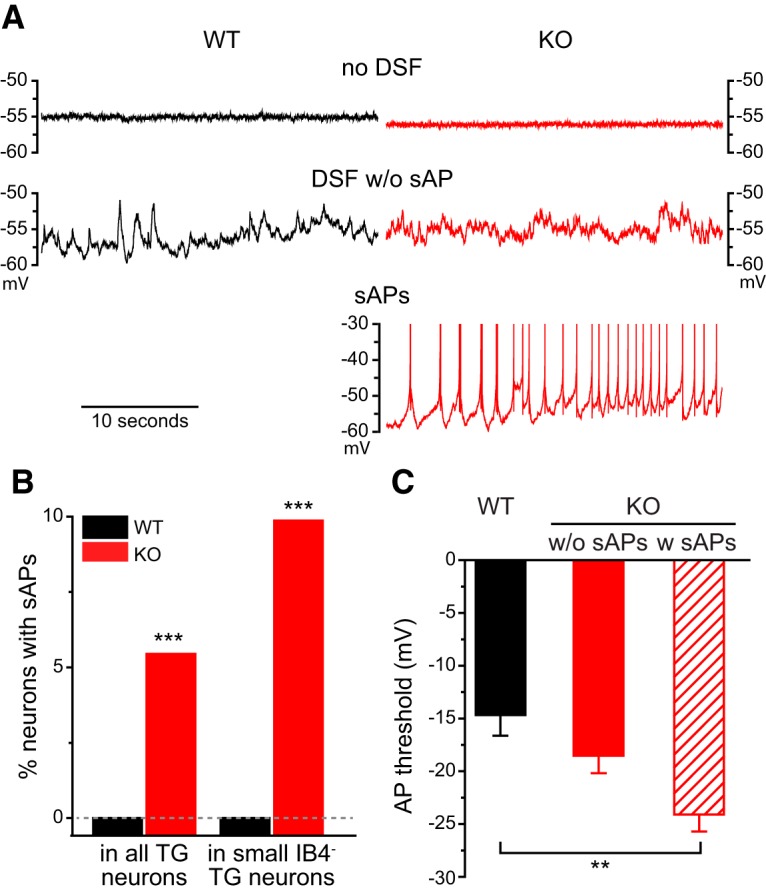
**Some small IB4^−^ TG neurons exhibit sAPs in the absence of TRESK. *A***, Representative traces of WT and TRESK KO small IB4^−^ TG neurons under current clamp recording without current injections. Top, Neurons exhibiting no DSF or sAPs; middle, neurons with DSF but without sAP; bottom, a KO neuron with sAPs. Note that every DSF results in an AP. ***B***, The percentage of neurons with sAPs in all TG neurons and in small IB4^−^ TG neurons from WT and TRESK KO mice, respectively. ***p < 0.001, Fisher’s exact test between the corresponding WT and KO groups. The dashed line indicates 0%. ***C***, The AP threshold of WT and KO small IB4^−^ TG neurons without sAPs (w/o sAPs; same neurons as in Fig. 3*B*) as well as KO TG neurons with sAPs (w sAPs; *n* = 11). **p < 0.01, one-way ANOVA with *post hoc* Bonferroni test.

Of all the WT TG neurons we recorded (*n* = 214), none showed sAP at *V*_rest_. On the contrary, 11 TRESK KO TG neurons exhibited sAPs at *V*_rest_ (Fig. 4*A*), all of which belonged to the small IB4^−^ subpopulation. This accounted for 5.6% (11 of 197) of the total KO TG neurons and 9.8% (11 of 112) of KO small IB4^−^ TG neurons that we recorded (Fig. 4*B*); again indicating that loss of TRESK preferentially increases the intrinsic excitability of small IB4^−^ TG neurons. Compared with WT small IB4^−^ TG neurons, KO neurons with sAPs had a similar *V*_rest_ (−48 ± 2 vs −51 ± 1 mV) but a considerably lower AP threshold (Fig. 4*C*), suggesting that TRESK activity may prevent sAPs through delaying the activation of voltage-gated Na^+^ channels. We also compared the incidence of depolarizing spontaneous fluctuation (DSF; Odem et al., 2018) in 16 WT and 21 KO small IB4^−^ TG neurons that had longer than 3 min gap-free current clamp recordings at *V*_rest_. In the WT group, 44% (7 of 16) neurons exhibited DSFs and none led to sAP. In the KO group, 38% of neurons either had DSFs without sAP (14%, 3 of 21) or had DSF with sAPs (24%, 5 of 21). The incidence of DSF was comparable between the KO and WT groups (*p* = 0.7, Fisher’s exact test). The amplitude of DSFs was also similar between WT and KO neurons (1.5–5 mV). Interestingly, in the five KO neurons with sAPs, every DSF was followed by a sAP (Fig. 4*A*), suggesting that endogenous TRESK activity plays crucial role in counterbalancing DSF and prevent sAPs in these neurons.

We also tested whether TG neuronal excitability was altered in TRESK HET mice. Despite the reduction of both TRESK and total persistent outward currents (Fig. 2*B*–*D*), neither small IB4^−^ nor IB4^+^ TG neurons from HET mice exhibited any changes of the measured passive and active electrophysiological properties (Fig. 3*B*–*E*; Table 1). This is consistent with our previous finding that only a substantial reduction of the endogenous TRESK activity results in hyper-excitation of TG neurons; and a moderate decrease of TRESK current is well tolerated (Guo et al., 2014).

**Figure 5.**
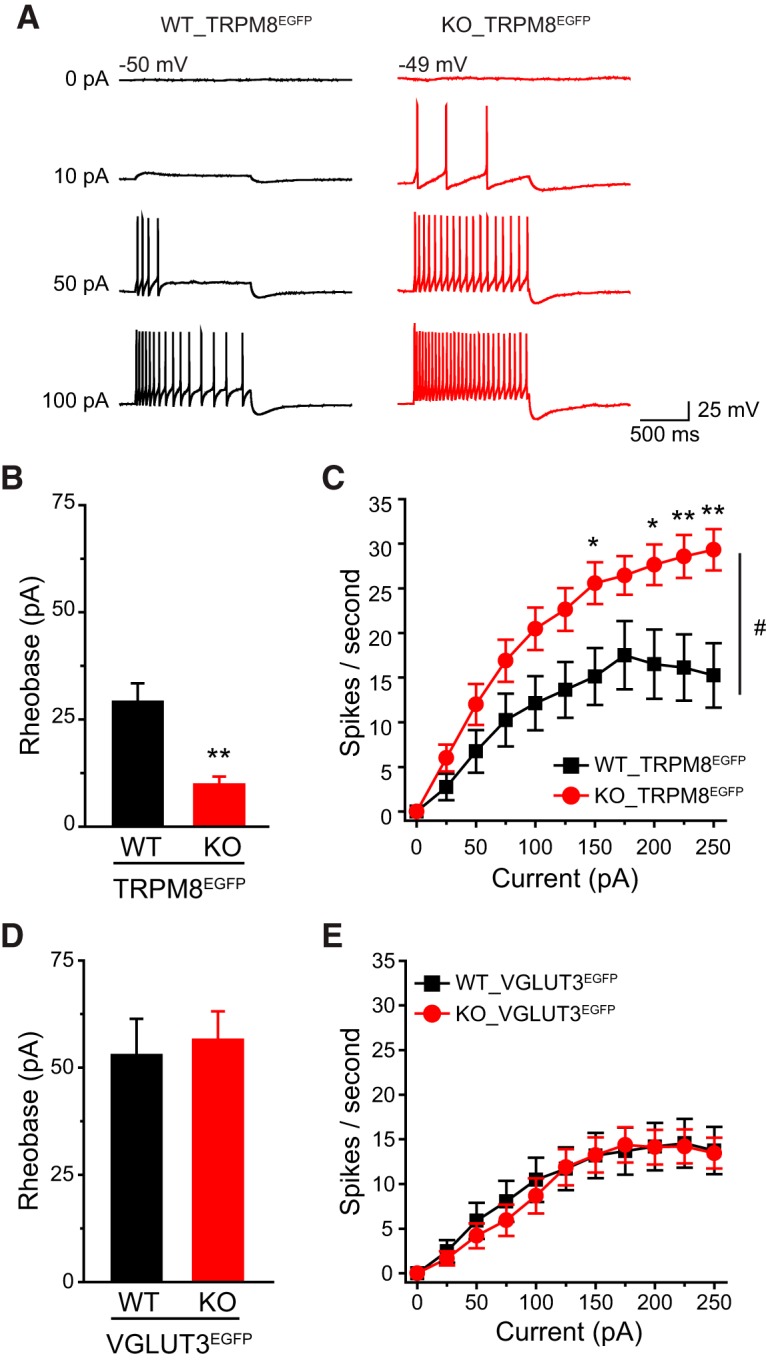
**Loss of TRESK increases the excitability of small IB4^−^ TG neurons expressing TRPM8 but not those expressing VGLUT3. *A***, Representative traces of APs generated by incremental depolarizing current injections in TG neurons from WT_TRPM8^EGFP^ and KO_TRPM8^EGFP^ mice. The values of *V*_rest_ and the current amplitude are indicated. ***B***, Mean rheobase of EGFP^+^ TG neurons from WT_TRPM8^EGFP^ and KO_TRPM8^EGFP^ mice (*n* = 14 and 18 neurons, respectively). **p < 0.01; two-tailed *t* test. ***C***, The spike frequency in response to incremental depolarizing current injections in EGFP^+^ TG neurons from WT_TRPM8^EGFP^ and KO_TRPM8^EGFP^ mice (same neurons as in ***C***). #*p* < 0.05; two-way RM ANOVA. *p < 0.05, **p < 0.01; two-tailed *t* test between the corresponding WT and KO groups. ***D***, ***E***, Mean rheobase and spike frequency of EGFP^+^ TG neurons from WT_VGLUT3^EGFP^ and KO_VGLUT3^EGFP^ mice (*n* = 19 and 27 neurons, respectively).

In a control experiment, we confirmed that 73% (459 of 626 neurons from 3 WT mice) of small IB4^−^ TG neurons in our culture contained CGRP-IR, indicating that the results obtained from small IB4^−^ TG neurons largely reflected changes consistent with how loss of TRESK affects the excitability of CGRP-expressing neurons within this subpopulation. However, the small IB4^−^ TG subpopulation also consists of neurons that express the cold sensor TRPM8 channels and C-LTMRs that express VGLUT3, respectively (Le Pichon and Chesler, 2014; Usoskin et al., 2015). These neurons were likely under-sampled when we recorded from small IB4^−^ TG neurons, because they each accounted for <20% of small IB4^−^ TG neurons. To identify these neurons in TG culture, we generated WT_TRPM8^EGFP^ and KO_TRPM8^EGFP^ mice that exhibit EGFP signal in TRPM8-expressing neurons (Dhaka et al., 2008). Indeed, only 11% (268 of 2365 neurons from 5 mice) of small IB4^−^ TG neurons were EGFP-positive (EGFP^+^) in cultures from WT_TRPM8^EGFP^ mice. Compared with their WT counterparts, the EGFP^+^, TRPM8-expressing TG neurons from TRESK KO mice showed a 70% reduction of rheobase and a much higher spike frequency in response to suprathreshold current injection (Fig. 5*A*–*C*). In WT EGFP^+^ neurons, the spike frequency plateaued in response to depolarizing current injections between 150 pA and 250 pA, whereas the spike frequency in KO EGFP^+^ neurons continued to increase at this range (Fig. 5*C*). We also generated WT_VGLUT3^EGFP^ and KO_VGLUT3^EGFP^ mice that exhibit EGFP fluorescence in VGLUT3-expressing neurons (Seal et al., 2009). Loss of TRESK had no effect on the excitability of EGFP^+^, VGLUT3-expressing C-LTMRs (Fig. 5*D*,*E*). Collectively, these results indicate that loss of TRESK in all TG neurons preferentially increases the intrinsic excitability of small IB4^−^ TG nociceptors expressing TRPM8 and likely those that express CGRP.

### Loss of TRESK enhances trigeminal nociception across multiple modalities in mice

**Figure 6.**
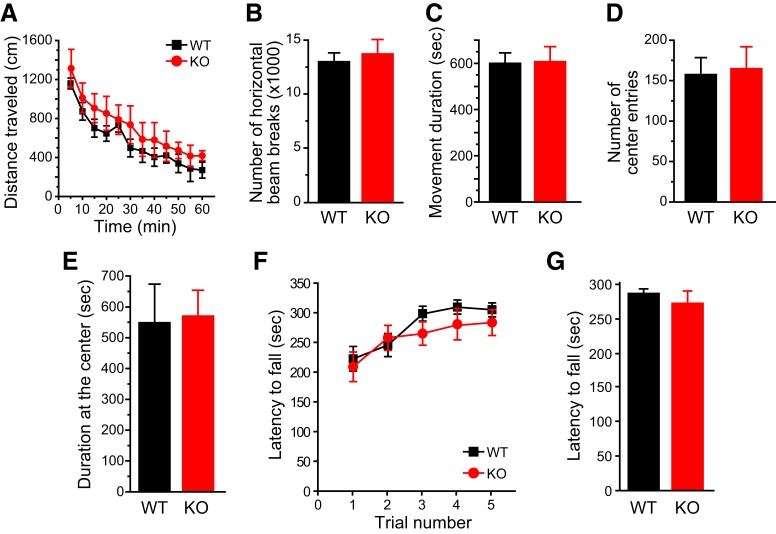
**Normal performance of TRESK KO mice in open field and rotarod tests. *A***, Distance traveled in the open field in 5 min bins (*n* = 5 adult female and 5 adult male mice in each group). ***B***–***E***, Total number of horizontal beam breaks (***B***), total duration of movement (***C***), total number of entries into the center square (***D***), and total time spent in the center square (***E***) during the 60 min testing period (same mice as in ***A***). ***F***, Latency to fall from the accelerating rotarod in five consecutive trials (*n* = 8 adult female and 7 adult male mice in each group). ***G***, The latency to fall from the accelerating rotarod was averaged from the five trials in individual mice (same mice as in ***F***).

We went on to investigate how TRESK KO mice respond to noxious stimuli on the face. TRESK KO mice were grossly normal. Both male and female KO mice gained weight normally. The performance of WT and KO mice did not differ in open field and rotarod tests (Fig. 6*A*–*G*), indicating that loss of TRESK does not affect general locomotion, coordination or the level of anxiety in mice. This allowed us to compare the nociceptive responses of WT and TRESK KO mice in a battery of pain models.

**Figure 7.**
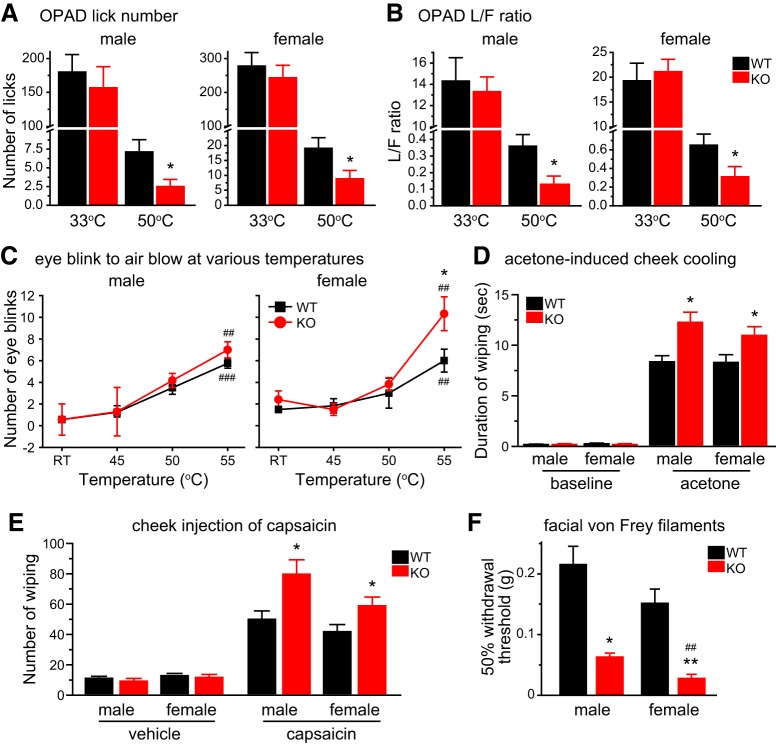
**Loss of TRESK enhances trigeminal nociception across multiple modalities. *A***, Average number of reward licks with cheeks pressing the Peltier bars set at 33°C or 50°C during a 3 min testing period in the OPAD assay (*n* = 7–11 mice in each group). *p < 0.05; two-tailed *t* test between the corresponding WT and KO groups. ***B***, Average L/F ratio (the number of reward licks per cheek contact) during a 3 min testing period in the OPAD assay (same mice as in ***A***). ***C***, The number of eye blinks evoked by blowing air at various temperatures to the eye for 10 s (*n* = 6–8 mice in each group). *p < 0.05; two-tailed *t* test between the corresponding WT and KO groups. ##*p* < 0.01, ###*p* < 0.001; one-way RM ANOVA with *post hoc* Bonferroni test compared with the corresponding RT groups. ***D***, Time spent wiping the treated area after application of 15 µl acetone to the cheek (*n* = 10–12 mice in each group). Baseline activity was recorded for 1 min in individual mice before acetone application. *p < 0.05; two-tailed *t* test between the corresponding WT and KO groups. ***E***, The number of forepaw wiping of the treated area after intradermal injection of 20 µl vehicle (saline with 1% DMSO) or capsaicin (1 µg in 20 µl vehicle) to the cheek (*n* = 7–13 mice in each group). *p < 0.05; two-tailed *t* test between the corresponding WT and KO groups. ***F***, The 50% withdrawal thresholds to punctate mechanical stimuli on forehead skin (*n* = 6–8 mice in each group). Kruskal–Wallis ANOVA on ranks with Tukey’s *post hoc* pairwise comparison: *p < 0.05, **p < 0.01; between the corresponding WT and KO groups; ##*p* < 0.01, between male and female KO groups.

We used an operant assay to assess the responses to noxious heat stimuli on the mouse facial skin. Mice needed to press their cheeks onto the Peltier bars set at various temperatures in the OPAD to lick the sweetened milk (the reward). In WT mice of either sex, both the total number of reward licking and the number of licks per facial contact (the L/F ratio) were greatly reduced when the bar temperature was increased from 33°C to 50°C (Fig. 7*A*,*B*, black bars). TRESK KO mice responded similarly to WT mice at 33°C (Fig. 7*A*,*B*, red bars), indicating that they were not impaired in learning and performing the operant assay. However, the total number of licks and the L/F ratio at 50°C were significantly lower in both male and female KO mice relative to WT controls (Fig. 7*A*,*B*), indicating that loss of TRESK increases the sensitivity and/or reduces the tolerance to noxious heat stimuli on facial skin. Interestingly, in female but not male TRESK KO mice, the number hot air (55°C) -evoked eye blinks was significantly higher than WT controls (Fig. 7*C*).

To test cold sensitivity, we reduced the facial skin temperature by applying acetone to the mouse cheek. The duration of wiping the treated area was significantly prolonged in KO mice than in WT controls (Fig. 7*D*), indicating a hyper-responsiveness to acetone evaporation-induced cooling. To measure facial responses to chemical stimuli, we injected capsaicin intradermally into the mouse cheek and found that the number of cheek wiping was significantly increased in both male and female KO mice compared with WT controls (Fig. 7*E*). Regarding facial mechanical sensitivity, we measured the threshold for evoking a withdrawal reflex to the application of von Frey filaments on mouse forehead skin. The 50% withdrawal threshold of TRESK KO mice was significantly lower than that of WT mice (Fig. 7*F*). Notably, the withdrawal threshold of female KO mice was even lower than that of male KO, although WT mice did not show sex difference in mechanical thresholds (*p* = 0.32, Fig. 7*F*). Together, these results indicate that endogenous TRESK activity inhibits the transmission and production of facial pain evoked by thermal, cold, chemical, and punctate mechanical stimuli.

### TRESK KO mice are hyper-responsive to stimuli that generate headache-related behaviors

**Figure 8.**
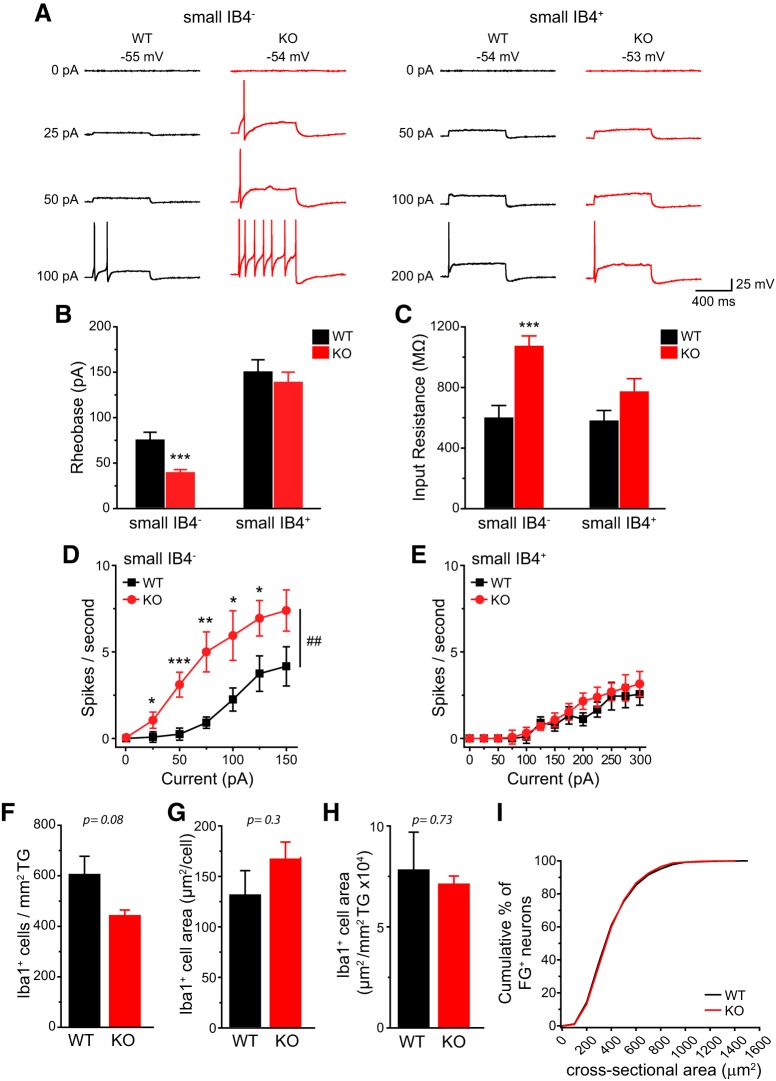
**The small IB4^−^ dural afferent neurons from TRESK KO mice show higher intrinsic excitability. *A***, Representative traces of APs generated by incremental depolarizing current injections in FG^+^ dural afferent neurons from WT and TRESK KO mice. The values of *V*_rest_ and the current amplitude are indicated. ***B***, ***C***, Mean rheobase (***B***) and R_in_ (***C***) of FG^+^ small IB4^−^ and IB4^+^ dural afferent neurons from WT and KO mice (*n* = 13–20 neurons in each group; for details of the intrinsic properties of dural afferent neurons, see Table 2). ***p < 0.001; two-tailed *t* test between the corresponding WT and KO groups. ***D***, ***E***, Input/output plots of the spike frequency in response to incremental depolarizing current injections in small IB4^−^ (***D***) and small IB4^+^ (***E***) dural afferent neurons from WT and KO mice (same neurons as in ***B***). ##*p* < 0.01; two-way RM ANOVA. *p < 0.05, **p < 0.01, ***p < 0.001; two-tailed *t* test between the corresponding WT and KO groups. ***F***, The density of Iba1^+^ macrophages in WT and KO TG (*n* = 3 mice in each group). ***G***, The mean area of individual Iba1^+^ macrophages in WT and KO TG (same mice as in ***F***). ***H***, The area of Iba1^+^ macrophages per mm^2^ TG in WT and KO mice (same mice as in ***F***). ***I***, Cumulative distributions of the cross-sectional areas of FG^+^ dural afferent neurons in WT and KO mice (*n* = 3627 and 3332 FG^+^ neurons pooled from 4 WT and 4 KO mice, respectively).

Does genetic loss of TRESK affect the excitability of dural afferent neurons, the primary sensory neurons in the trigeminovascular pathway subserving headache? To address this question, we used FG to retrogradely label dural afferent neurons in adult mice. The size distribution of FG-labeled (FG^+^) dural afferent neurons was similar between WT and TRESK KO mice (Fig. 8*I*). The small IB4^−^ dural afferent neurons from KO mice exhibited a significantly higher *R*_in_ and a >50% reduction of rheobase compared with their WT counterparts (Fig. 8*A*–*C*). The number of APs evoked by depolarizing current injections was significantly increased in this subpopulation of KO dural afferent neurons relative to WT controls (Fig. 8*D*). On the contrary, the rheobase, *R*_in_ and spike frequency were similar in small IB4^+^ dural afferent neurons from WT and KO mice (Fig. 8*A*–*C*,*E*). The values of *V*_rest_, AP threshold, amplitude, half-width, and AHP amplitude were all comparable between WT and KO FG^+^ neurons (Table 2). We conclude that loss of TRESK activity selectively increases the intrinsic excitability of small IB4^−^ dural afferent neurons.

**Table 2.** Intrinsic properties of FG-labeled dural afferent neurons from adult WT and TRESK KO mice

	Diameter, μm	Capacitance, pF	*R*_in_, MΩ	*V*_rest_, mV	Rheobase, pA	AP threshold, mV	AP amplitude, mV	AP half-width, ms	AHP amplitude, mV	Cell number
Small IB4-negative dural afferent neurons										
WT	19.2 ± 0.5	20.9 ± 1.1	586 ± 84	−55.6 ± 1.2	73 ± 9	−16.7 ± 1.6	113.0 ± 2.7	3.3 ± 0.4	−16.9 ± 1.3	15
KO	18.2 ± 1.3	20.3 ± 1.1	1058 ± 60***	−55.5 ± 1.6	38 ± 4***	−14.6 ± 1.1	111.5 ± 1.7	4.0 ± 0.3	−18.6 ± 1.4	20
Small IB4-positive dural afferent neurons										
WT	20.4 ± 0.7	21.7 ± 1.5	565 ± 71	−54.4 ± 1.0	150 ± 14	−14.9 ± 1.3	115.9 ± 2.9	4.6 ± 0.5	−17.1 ± 1.4	13
KO	17.7 ± 1.0	21.2 ± 1.3	757 ± 89	−55.3 ± 1.5	138 ± 11	−15.3 ± 1.7	113.4 ± 4.6	4.9 ± 0.5	−18.6 ± 1.6	20

***p < 0.001; two-tailed *t* test between the corresponding WT and KO groups.

In a mouse model of familial hemiplegic migraine type 1, the number of macrophages was significantly increased in TG, which may contribute to the headache pathophysiology (Franceschini et al., 2013). We stained adult WT and TRESK KO TG sections with an antibody recognizing Iba1, a macrophage marker. Both the density and the cross-sectional area of Iba1^+^ cells were similar between WT and KO TG (Fig. 8*F*–*H*), suggesting that loss of TRESK does not significantly affect resident macrophages in TG.

**Figure 9.**
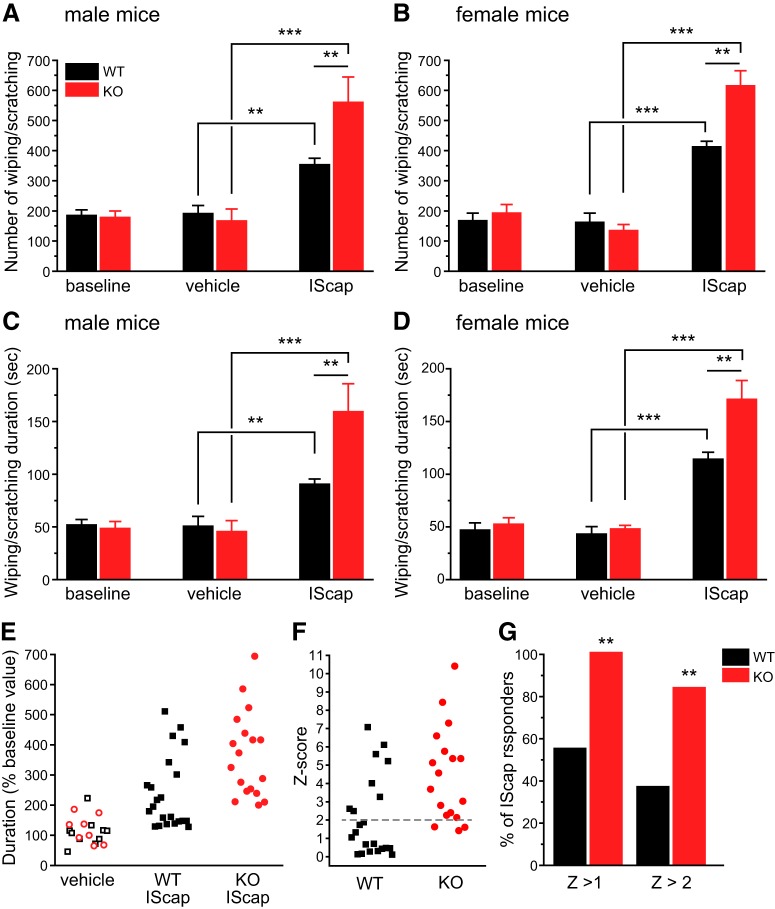
**TRESK KO mice are hyper-responsive to dural application of IScap. *A***, The number of V1-directed forepaw wiping and hindpaw scratching within the 2 h recording period in male mice. Baseline behaviors were recorded in all mice 7 d postsurgery. The next day, mice received dural application of vehicle (*n* = 5 WT and 4 KO mice) or IScap (*n* = 10 WT and 8 KO mice). The behaviors were recorded 0.5–2.5 h after the dural application. **p < 0.01, ***p < 0.001; two-way ANOVA with *post hoc* Bonferroni test. ***B***, The number of V1-directed behaviors in female mice (*n* = 5 WT and 4 KO mice in the vehicle groups; *n* = 12 WT and 10 KO mice in the IScap groups). ***C***, ***D***, The duration of V1-directed forepaw wiping and hindpaw scratching within the 2 h recording period in male (***C***) and female (***D***) mice (same mice as in ***A*** and ***B***). ***E***, Scatter plots of the duration of V1-directed behavior (normalized to the baseline values in individual mice) in response to dural application of vehicle or IScap. Data from the male and female mice are combined. Data from vehicle-treated mice are pooled together (open black square and red circle indicate WT and KO mice, respectively). ***F***, The *Z* score of individual IScap-treated mice, calculated as *Z* = (value – mean)/SD. Value is the normalized duration of individual IScap-treated mice in ***E***. Mean and SD are calculated from the normalized duration of all vehicle-treated mice in ***E***. ***G***, The percentage of WT and KO mice that respond to dural application of IScap, using *Z* > 1 and *Z* > 2 (> mean duration + 1 × SD or + 2 × SD of vehicle-treated mice) as the threshold respectively. **p < 0.01, Fisher’s exact test between the corresponding WT and KO groups.

Next, we investigated whether endogenous TRESK activity regulates the generation of headache-related behaviors in mice (Huang et al., 2016). After recovery from craniectomy for 7 d, both WT and TRESK KO mice exhibited some spontaneous forepaw wiping and hindpaw scratching behavior within the trigeminal V1 dermatome during a 2 h observation period in the home cage (Fig. 9*A*–*D*, baseline groups). Dural application of 20 µl vehicle did not increase V1-directed behavior above the basal level in either WT or KO mice (Fig. 9*A*–*D*, vehicle groups). In WT mice, dural application of 20 µl IScap, which contained capsaicin and a mixture of inflammatory mediators, elicited more robust V1-directed wiping and scratching than vehicle treatment (Fig. 9*A*–*D*, black bars). Our previous study suggests that dural IScap-induced behavior are mechanistically related to the ongoing headache in humans, as they can be reduced to the control level by pretreatment of mice with anti-migraine drugs (Huang et al., 2016). Both the number (Fig. 9*A*,*B*) and the duration (Fig. 9*C*,*D*) of V1-directed behaviors were significantly higher in TRESK KO mice than in WT mice regardless of sex, indicating that loss of TRESK renders both male and female mice hyper-responsive to stimuli that evoke headache-related behaviors.

We went on to ask whether loss of TRESK alters the likelihood to develop headache-related behaviors in mice. To increase statistical power, we combined data from male and female mice and normalized the duration of vehicle- or IScap-induced behavior to the baseline values in individual mice (Fig. 9*E*). Because WT and KO mice responded similarly to vehicle application, we used all vehicle-treated mice as the reference population to calculate the *Z*-score (the number of SDs from the mean of all vehicle-treated mice) of each IScap-treated mouse (Fig. 9*F*). When we used *Z* > 2 as the threshold, <40% (8 of 22) of WT mice met the criteria as IScap responders (i.e. the duration of IScap-induced behavior was longer than the mean + 2 SD of the duration of vehicle-induced behavior), whereas >80% (15 of 18) TRESK KO mice were responders (Fig. 9*G*). Using a less stringent criteria (Z > 1), all KO mice responded to dural IScap but <60% of WT mice could be classified as responders (Fig. 9*G*). Collectively, these data suggest that loss of TRESK increases both the likelihood and the magnitude of headache-related behavior in response to IScap-evoked activation of the trigeminovascular pathway.

### Genetic loss of TRESK does not alter the excitability of DRG neurons or the behavioral responses to noxious stimuli on the hindpaw and in visceral tissues

**Figure 10.**
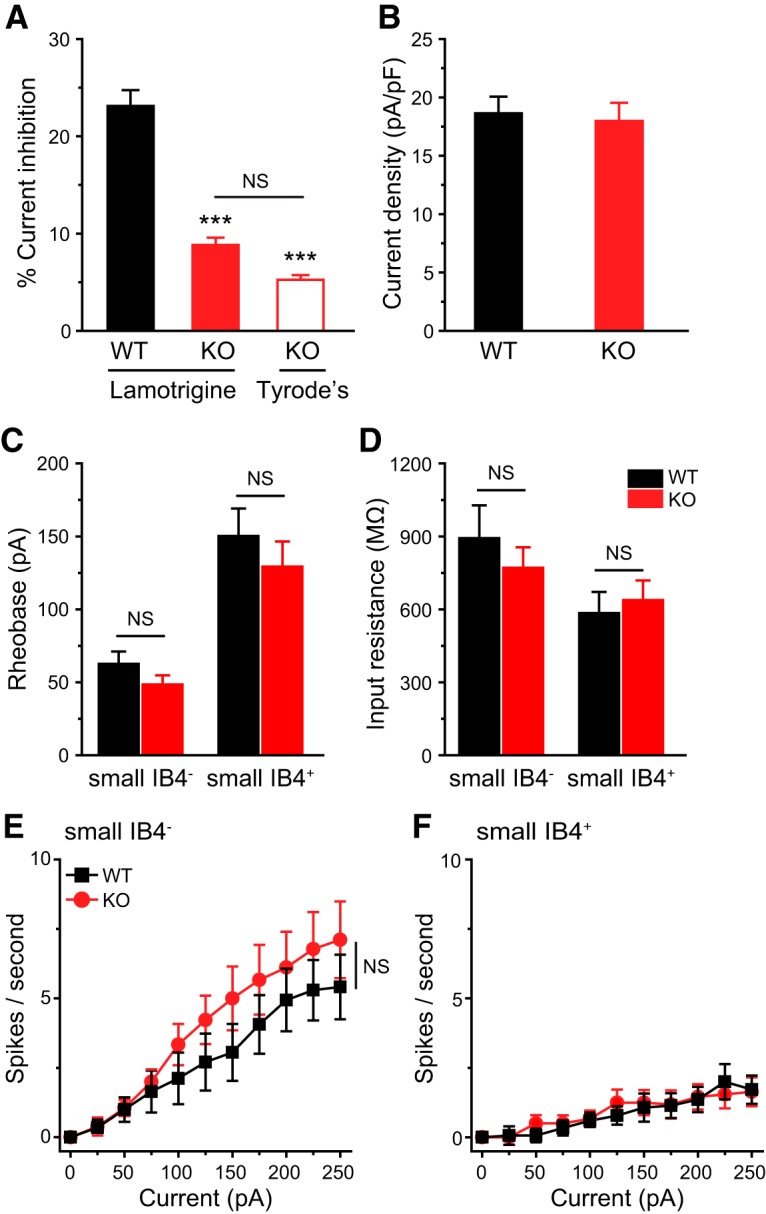
**Genetic loss of TRESK does not alter the excitability of small lumbar DRG neurons. *A***, ***B***, The percentage of lamotrigine-sensitive persistent K^+^ currents (***A***) and the total persistent outward current density (***B***) in small-diameter DRG neurons from adult WT and TRESK KO mice (*n* = 14–20 neurons in each group). ***p < 0.001; one-way ANOVA with *post hoc* Bonferroni test, compared with the WT group. NS, No statistically significant difference. ***C***, ***D***, Mean rheobase (***C***) and *R*_in_ (***D***) of small IB4^−^ and IB4^+^ DRG neurons from WT and TRESK KO mice (*n* = 16–19 neurons in each group; for details of the intrinsic properties of DRG neurons, see Table 3). ***E***, ***F***, Input/output plots of the spike frequency in response to incremental depolarizing current injections in small IB4^−^ (***E***) and small IB4^+^ (***F***) DRG neurons from WT and KO mice (same neurons as in ***C***).

Having established that genetic loss of TRESK selectively increases the excitability of TG nociceptors and small IB4^−^ dural afferent neurons, we proceeded to investigate whether the excitability of small-diameter DRG neurons were altered in TRESK KO mice. As in TG neurons, 30 µm of lamotrigine blocked 20–30% of persistent outward currents in every adult WT DRG neurons that we recorded but had no significant effect on currents in KO DRG neurons (Fig. 10*A*). Surprisingly, the size of total persistent outward current remained comparable between WT and KO DRG neurons (Fig. 10*B*), suggesting that genetic loss of endogenous TRESK current is fully compensated in DRG neurons. Consequently, in either small IB4^−^ or small IB4^+^ DRG subpopulation, WT and KO neurons exhibit similar *R*_in_, rheobase, spike frequency as well as other passive and active electrophysiological properties (Fig. 10*C*–*F*; Table 3). Together, we conclude that the impact of genetic loss of TRESK on total persistent outward currents and PAN excitability is cell-type-specific and is not determined by its expression pattern.

**Table 3.** Intrinsic properties of small-diameter DRG neurons from adult WT and TRESK KO mice

	Diameter, μm	Capacitance, pF	*R*_in_,MΩ	*V*_rest_, mV	Rheobase, pA	AP threshold, mV	AP amplitude, mV	AP half-width, ms	AHP amplitude, mV	Cell number
Small IB4-negative neurons										
WT	21.3 ± 0.5	22.0 ± 0.9	882 ± 135	−56.7 ± 1.2	61 ± 9	−15.8 ± 1.7	111.9 ± 3.0	4.7 ± 0.4	−18.4 ± 1.5	16
KO	20.5 ± 0.5	19.6 ± 1.1	761 ± 83	−55.9 ± 1.5	47 ± 6	−17.9 ± 2.0	115.2 ± 2.0	4.2 ± 0.4	−20.0 ± 1.2	18
Small IB4-positive neurons										
WT	21.3 ± 0.7	21.9 ± 1.2	575 ± 86	−57.7 ± 1.5	149 ± 19	−14.3 ± 2.0	115.4 ± 2.2	5.9 ± 0.6	−19.1 ± 1.2	17
KO	21.8 ± 0.7	24.3 ± 1.7	627 ± 81	−54.7 ± 1.1	128 ± 17	−11.4 ± 2.4	114.8 ± 2.9	5.7 ± 0.4	−21.9 ± 1.5	19

Our findings contradict with a previous study reporting a mild reduction of persistent outward currents and AP rheobase in DRG neurons from homozygous mice carrying a missense mutation that renders the TRESK channel nonfunctional (Dobler et al., 2007). Several differences between the two studies may contribute to the discrepancy. First, the persistent outward currents in DRG neurons may be compensated in the absence of TRESK protein but not in the presence of nonfunctional TRESK channels (Rossi et al., 2015). Unfortunately, it is not clear whether the mutation affects the expression level of TRESK subunits, the efficiency of dimerization, and/or the number of channels on the plasma membrane. Second, given that the rheobase of small IB4^−^ and IB4^+^ DRG neurons differs significantly even in WT DRG neurons (Fig. 10C), including all DRG neurons into one experimental group as in the previous study makes it difficult to interpret the results. Last, it is possible that results differ based on the duration of neurons are maintained *in vitro*. We recorded DRG neurons between 2 and 4 DIV, whereas in earlier work neurons were recorded between 11 and 14 DIV. It is reported that WT DRG neurons cultured for 5 DIV exhibit altered K^+^ channel expression and excitability (Martinez-Espinosa et al., 2015; Dawes et al., 2018).

**Figure 11.**
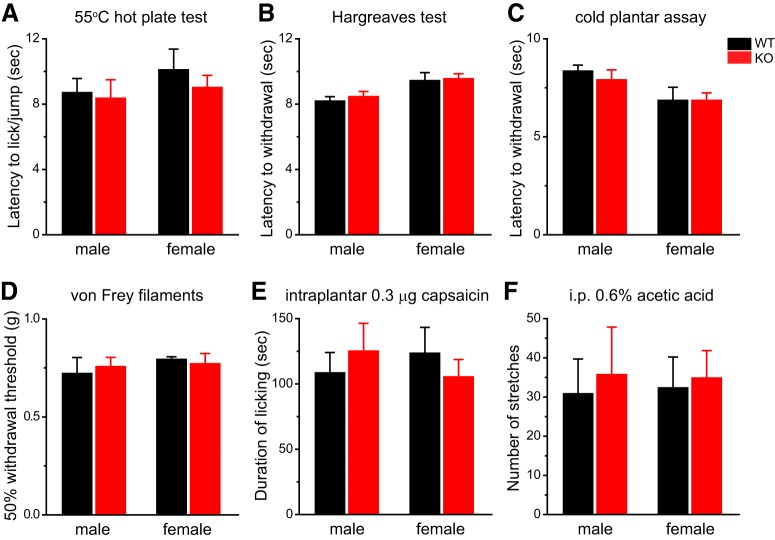
**Both male and female TRESK KO mice show normal responses to stimuli on the hindpaw and in visceral tissues. *A***, Licking/jump latency on the 55°C hot plate (*n* = 5–6 mice in each group). ***B***, Withdrawal latency to radiant heat stimuli on the hindpaw (*n* = 5–6 mice in each group). ***C***, Withdrawal latency to cold stimuli on the hindpaw (*n* = 5–12 mice in each group). ***D***, Withdrawal threshold of a mechanical stimulus on the hindpaw (von Frey hair; *n* = 5–6 mice in each group). The results were verified by another experimenter in a separate cohort of WT and KO littermates. ***E***, Duration of licking the hindpaw after intraplantar injection of 0.3 µg capsaicin (in 10 µl saline with 1% DMSO, *n* = 5–6) or (*n* = 5–6 mice in each group). Vehicle injection induced <5 s licking in individual mice. ***F***, The number of abdominal stretches produced by intraperitoneal injection of 0.6% acetic acid (*n* = 6–8 mice in each group).

We went on to investigate how TRESK KO mice respond to noxious stimuli on the hindpaw. Consistent with the results from DRG neurons *in vitro*, we found no difference between WT and KO mice in their responses to thermal and cooling stimuli to the hindpaw, regardless of sex (Fig. 11*A*–*C*). The 50% withdrawal threshold to von Frey filaments on the hindpaw was also comparable between WT and KO mice (Fig. 11*D*). Two previous studies reported that TRESK KO mice exhibited shorter response latency on a hot plate and a reduction of hindpaw mechanical threshold when housed individually (Chae et al., 2010; Castellanos et al., 2017). To verify our results, we compared the thermal responses between the group-housed WT and KO mice to 55°C hot plate as well as to radiant heat. In both assays, there was no difference between WT and KO mice in the latency to respond to heat stimuli (Fig. 11*A*,*B*). Likewise, another experimenter tested a separate cohort of WT and KO littermates for the 50% withdrawal threshold to von Frey filaments on the hindpaw and saw no difference between the groups (data not shown). In addition, WT and KO mice showed similar duration of licking in response to hindpaw injection of capsaicin (Fig. 11*E*). Last, we tested the mice in a model of acute inflammatory visceral pain. The number of abdominal stretches evoked by intraperitoneal injections of dilute acetic acid did not differ in the WT and KO mice (Fig. 11*F*). We conclude that genetic loss of TRESK does not alter the behavioral responses to noxious stimuli on the body and viscera in male and female mice regardless of the stimulus modality. This is consistent with the finding that loss of TRESK does not affect the intrinsic excitability of DRG neurons.

## Discussion

In this study, we investigated how genetic loss of TRESK affects PAN excitability and nociception. Consistent with previous work (Dobler et al., 2007; Yoo et al., 2009; Lafrenière et al., 2010; Guo and Cao, 2014; Kollert et al., 2015), we found that functional TRESK channels are present in all WT PANs. Loss of TRESK decreases the persistent outward current in all TG neurons. In small IB4^−^ TG nociceptors, this results in higher *R*_in_, lower rheobase, and increase in spike frequency, indicating that endogenous TRESK is activated around *V*_rest_ and during the weak depolarizations before AP initiation to reduce the membrane resistance and increase outward currents, thereby serving as a brake to oppose membrane depolarization and limit AP firing. Importantly, we observed sAPs at *V*_rest_ in 10% of KO neurons but not in any of the WT neurons. Numerous studies indicate that ongoing activity of PANs maintains the “spontaneous” pain under pathologic conditions (Gracely et al., 1992; Baron et al., 2013; Haroutounian et al., 2014). Our data suggest that TRESK dysfunction may contribute to the spontaneous pain resulting from the ongoing activity in small IB4^−^ TG nociceptors (Odem et al., 2018).

In small IB4^+^ TG neurons, loss of TRESK leads to a small reduction of rheobase, with no changes in spike frequency and no sAPs, indicating a limited role of TRESK in regulating the excitability of these neurons. This is in line with earlier studies identifying another K_2P_ channel TREK2 as well as Ca^2+^- and Na^+^-modulated K^+^ channels as the key regulators of the *V*_rest_ and intrinsic excitability of small IB4^+^ PANs (Zhang et al., 2010; Acosta et al., 2014; Martinez-Espinosa et al., 2015). In medium-sized TG neurons, although TRESK channels mediate ∼25% of the persistent outward current at −25 mV, the TRESK current density around *V*_rest_ is very small. This may explain why loss of TRESK has no effect on the passive membrane properties or the intrinsic excitability of these neurons at all. Collectively, the present data delineate a cell-type-specific role of TRESK channels in controlling TG neuronal excitability. Despite the presence of functional TRESK in all PANs, genetic loss of TRESK preferentially increases the excitability of small IB4^−^ TG nociceptors expressing TRPM8 and likely those that express CGRP as well. Notably, a moderate reduction of TRESK and total persistent outward currents in TRESK HET mice does not affect neuronal excitability, indicating that 50% of endogenous TRESK activity is sufficient to maintain normal intrinsic excitability and to prevent sAPs in TG neurons.

Consistent with the *in vitro* data, we made several key *in vivo* observations that endogenous TRESK activity regulates trigeminal nociception across various modalities in both male and female mice, with females being more sensitive than males to the loss of TRESK in some aspects of trigeminal nociception. First, the hyper-excitation of small IB4^−^ TG neurons, especially those expressing CGRP, likely accounts for the hypersensitivity of KO mice to facial thermal and chemical stimuli (McCoy et al., 2013). Second, the reduction of rheobase in small IB4^+^ TG neurons may underlie the hypersensitivity of facial skin to punctate mechanical stimuli in KO mice, as ablation of IB4^+^ DRG neurons expressing Mrgprd receptor reduces the responses of dorsal horn neurons and increases the withdrawal threshold to mechanical stimuli (Cavanaugh et al., 2009; Zhang et al., 2013). This also implicates that the responses to punctate mechanical stimuli are likely triggered by the spatial summation of single APs from multiple IB4^+^ fibers rather than by the temporal summation of multiple APs from the same fiber. Last, the response to acetone-induced cooling of facial skin is more robust in TRESK KO mice, likely resulting from the hyper-excitation of TRPM8-expressing TG neurons (Bautista et al., 2007; Dhaka et al., 2007; Knowlton et al., 2013; Pogorzala et al., 2013). This indicates that endogenous TRESK, along with other K_2P_ channels including TREK1, TREK2, and TASK3 (Viana et al., 2002; Madrid et al., 2009; Noël et al., 2009; Vetter et al., 2013; Morenilla-Palao et al., 2014; Pereira et al., 2014), acts as an excitability brake in TRPM8-expressing neurons to modulate the sensitivity to cooling/cold stimuli.

Most importantly, we found that loss of TRESK increases the excitability of small IB4^−^ dural afferent neurons. TRESK KO mice are more responsive to the dural application of IScap, with more robust headache-related behaviors compared with WT controls. This suggests that the endogenous TRESK activity negatively regulates the trigeminovascular pathway and prevents the onset of headache, likely through controlling the excitability of small IB4^−^ dural afferent neurons. Future study is warranted to determine whether common migraine triggers precipitate headache episodes through a reduction of TRESK activity in small IB4^−^ dural afferent neurons.

Although TRESK mRNA is highly expressed in human TG neurons and TRESK mutations are identified in migraine patients (Lafrenière et al., 2010; Andres-Enguix et al., 2012; Rainero et al., 2014; Flegel et al., 2015; LaPaglia et al., 2018), the causal relationship between TRESK function and migraine susceptibility is not established. A recent study reports that expression of migraine-associated dominant-negative TRESK subunits does not alter the excitability of small TG neurons (Royal et al., 2019). It is suggested that the dysfunction of K_2P_ TREK1/2 channels, and not of TRESK alone, contributes to the increase in TG neuronal excitability and alters the pain processing in migraine patients with frameshift TRESK mutations (Royal et al., 2019). Unfortunately, to what extent mutant TRESK subunits affect the TRESK current, the total persistent outward current and the excitability of dural afferent neurons are not shown. More importantly, including all small TG neurons into one group may mask the effects of mutant subunits on the excitability of TG subpopulation(s), as indicated by our results. We also find that a mild reduction of TRESK activity does not alter the excitability of TG neurons. That said, what we have observed in TRESK KO mice strongly suggest that a profound reduction of TRESK activity will significantly increase the excitability of small IB4^−^ dural afferent neurons and enhance the activation of the trigeminovascular pathway, leading to a higher susceptibility to headache episodes.

Unlike TG neurons, DRG neurons fully compensate for the genetic loss of TRESK, showing no changes of total persistent outward current or the intrinsic excitability. This likely results from the upregulation of other K^+^ channels that are active at *V*_rest_. A comparison of the transcriptomes from mouse TG and DRG neurons reveals that, despite being overwhelmingly similar, 15 Hox family of transcription factors are exclusively expressed in adult DRG neurons (Lopes et al., 2017). Whether this provides a possible molecular basis for the differential adaptive changes in TRESK KO DRG and TG neurons merits further investigation.

In line with the *in vitro* results, multiple behavioral tests reveal no difference between WT and TRESK KO mice in their responses to various noxious stimuli on the hindpaw and in viscera. This recapitulates the clinical presentations of human genetic TRESK dysfunction, as dominant-negative TRESK mutations are associated with migraine but not with body or visceral pain in humans. In light of previous reports that TRESK KO mice are hypersensitive to thermal and mechanical stimuli on the hindpaw (Chae et al., 2010; Castellanos et al., 2017), we verified our results with two thermal nociceptive assays and with mechanical threshold measurements in two cohorts of mice by two experimenters. The discrepancy may arise from differences between genetic background (C57BL/6J versus C57BL/6N, plus the genetic drift in individual colonies), housing conditions (group-housed vs individually-housed) and diet composition etc. If this is the case, it will suggest that nature and/or nurture influence how genetic TRESK dysfunction affect body/visceral nociception. Our results suggest that, for some individuals with certain genetic compositions and raised in certain environment, their DRG neurons may tolerate the genetic TRESK dysfunction and maintain normal intrinsic excitability, resulting in normal body/visceral nociception as adults.

Earlier studies clearly indicate that reduction of TRESK activity in adult mice increases the sensitivity to mechanical stimuli on the hindpaw (Bautista et al., 2008; Lennertz et al., 2010; Tulleuda et al., 2011; Zhou et al., 2016). Our results complement rather than contradict these findings. Together, they suggest the model that DRG neurons can compensate for the loss of TRESK during development but not the reduction of TRESK activity in adulthood. Admittedly, our findings in the TRESK global KO mice do not exclude a role for TRESK outside PANs in trigeminal pain regulation. The consistency between the electrophysiology data from PANs *in vitro* and the results from *in vivo* behavioral tests strongly suggest that endogenous TRESK regulate trigeminal nociception mainly through controlling the excitability of TG nociceptors.

In conclusion, our study highlights some exquisite differences between TG and DRG neurons in response to ion channel defects. We provide evidence that genetic loss of TRESK in all PANs preferentially increases the excitability of small-diameter TG nociceptors and enhances trigeminal nociception. Importantly, our results indicate that genetic loss of TRESK significantly increases the likelihood of developing headache. This establishes a foundation for further elucidating the unique molecular and cellular basis of trigeminal pain, especially migraine headache.

## Synthesis

Reviewing Editor: Douglas Bayliss, University of Virginia School of Medicine Decisions are customarily a result of the Reviewing Editor and the peer reviewers coming together and discussing their recommendations until a consensus is reached. When revisions are invited, a fact-based synthesis statement explaining their decision and outlining what is needed to prepare a revision will be listed below. Note: If this manuscript was transferred from JNeurosci and a decision was made to accept the manuscript without peer review, a brief statement to this effect will instead be what is listed below.

This paper makes an important contribution to understanding the role of TRESK in TG vs DRG sensory neurons, with potential implications for how TRESK mutations in humans may lead specifically to migraine, but not other pain disorders. The authors have adequately responded to previous reviewer comments, and make a reasonable case that it is beyond the scope of this paper to identify the nature of compensatory currents in DRG neurons or to account for discrepancies with earlier studies with another strain of these knockout mice. Whereas those differences between labs/strains raise concerns about the robustness of results with these TRESK KO mice, the current paper appears technically solid and it is therefore of value to share these results with the community.

I would recommend one further change to the text:

l. 528 in title: “Loss of TRESK enhances trigeminal nociception across all modalities in mice”; change “across all” to “across multiple”

## References

[B1] Acosta C, Djouhri L, Watkins R, Berry C, Bromage K, Lawson SN (2014) TREK2 expressed selectively in IB4-binding C-fiber nociceptors hyperpolarizes their membrane potentials and limits spontaneous pain. J Neurosci 34:1494–1509. 10.1523/JNEUROSCI.4528-13.2014 24453337 PMC3898302

[B2] Akiyama T, Carstens MI, Carstens E (2010) Differential itch- and pain-related behavioral responses and micro-opoid modulation in mice. Acta Derm-Venereol 90:575–581. 10.2340/00015555-0962 21057739

[B3] Anderson EM, Mills R, Nolan TA, Jenkins AC, Mustafa G, Lloyd C, Caudle RM, Neubert JK (2013) Use of the operant orofacial pain assessment device (OPAD) to measure changes in nociceptive behavior. J Vis Exp 76:e50336 10.3791/50336 PMC3727247 23792907

[B4] Andres-Enguix I, Shang L, Stansfeld PJ, Morahan JM, Sansom MS, Lafrenière RG, Roy B, Griffiths LR, Rouleau GA, Ebers GC, Cader ZM, Tucker SJ (2012) Functional analysis of missense variants in the TRESK (KCNK18) K channel. Sci Rep 2:237. 10.1038/srep00237 22355750 PMC3266952

[B5] Bae JY, Kim JH, Cho YS, Mah W, Bae YC (2015) Quantitative analysis of afferents expressing substance P, calcitonin gene-related peptide, isolectin B4, neurofilament 200, and Peripherin in the sensory root of the rat trigeminal ganglion. J Comp Neur 523:126–138. 10.1002/cne.23672 25185935

[B6] Baron R, Hans G, Dickenson AH (2013) Peripheral input and its importance for central sensitization. Ann Neurol 74:630–636. 10.1002/ana.24017 24018757

[B7] Bautista DM, Siemens J, Glazer JM, Tsuruda PR, Basbaum AI, Stucky CL, Jordt SE, Julius D (2007) The menthol receptor TRPM8 is the principal detector of environmental cold. Nature 448:204–208. 10.1038/nature05910 17538622

[B8] Bautista DM, Sigal YM, Milstein AD, Garrison JL, Zorn JA, Tsuruda PR, Nicoll RA, Julius D (2008) Pungent agents from Szechuan peppers excite sensory neurons by inhibiting two-pore potassium channels. Nat Neurosci 11:772–779. 10.1038/nn.2143 18568022 PMC3072296

[B9] Brenner DS, Golden JP, Gereau RW (2012) A novel behavioral assay for measuring cold sensation in mice. PloS One 7:e39765. 10.1371/journal.pone.0039765 22745825 PMC3382130

[B10] Cao YQ, Mantyh PW, Carlson EJ, Gillespie AM, Epstein CJ, Basbaum AI (1998) Primary afferent tachykinins are required to experience moderate to intense pain. Nature 392:390–394. 10.1038/32897 9537322

[B11] Castellanos A, Andres A, Bernal L, Callejo G, Comes N, Gual A, Giblin JP, Roza C, Gasull X (2017) Pyrethroids inhibit K2P channels and activate sensory neurons: basis of insecticide-induced paraesthesias. Pain 159:92-105. 10.1097/j.pain.0000000000001068 PMC5737456 28937579

[B12] Cavanaugh DJ, Lee H, Lo L, Shields SD, Zylka MJ, Basbaum AI, Anderson DJ (2009) Distinct subsets of unmyelinated primary sensory fibers mediate behavioral responses to noxious thermal and mechanical stimuli. Proc Natl Acad Sci U S A 106:9075–9080. 10.1073/pnas.0901507106 19451647 PMC2683885

[B13] Chae YJ, Zhang J, Au P, Sabbadini M, Xie GX, Yost CS (2010) Discrete change in volatile anesthetic sensitivity in mice with inactivated tandem pore potassium ion channel TRESK. Anesthesiology 113:1326–1337. 10.1097/ALN.0b013e3181f90ca5 21042202 PMC3010361

[B14] Chaplan SR, Bach FW, Pogrel JW, Chung JM, Yaksh TL (1994) Quantitative assessment of tactile allodynia in the rat paw. J Neurosci Methods 53:55–63. 7990513 10.1016/0165-0270(94)90144-9

[B15] Choi JS, Dib-Hajj SD, Waxman SG (2007) Differential slow inactivation and use-dependent inhibition of Nav1.8 channels contribute to distinct firing properties in IB4+ and IB4- DRG neurons. J Neurophysiol 97:1258–1265. 10.1152/jn.01033.2006 17108087

[B16] Constandil L, Goich M, Hernández A, Bourgeais L, Cazorla M, Hamon M, Villanueva L, Pelissier T (2012) Cyclotraxin-B, a new TrkB antagonist, and glial blockade by propentofylline, equally prevent and reverse cold allodynia induced by BDNF or partial infraorbital nerve constriction in mice. J Pain 13:579–589. 10.1016/j.jpain.2012.03.008 22560237

[B17] Czirják G, Toth ZE, Enyedi P (2004) The two-pore domain K+ channel, TRESK, is activated by the cytoplasmic calcium signal through calcineurin. J Biol Chem 279:18550–18558. 10.1074/jbc.M312229200 14981085

[B18] Dawes JM, Weir GA, Middleton SJ, Patel R, Chisholm KI, Pettingill P, Peck LJ, Sheridan J, Shakir A, Jacobson L, Gutierrez-Mecinas M, Galino J, Walcher J, Kühnemund J, Kuehn H, Sanna MD, Lang B, Clark AJ, Themistocleous AC, Iwagaki N, et al. (2018) Immune or genetic-mediated disruption of CASPR2 causes pain hypersensitivity due to enhanced primary afferent excitability. Neuron 97:806–822.e810. 10.1016/j.neuron.2018.01.033 29429934 PMC6011627

[B19] Dhaka A, Earley TJ, Watson J, Patapoutian A (2008) Visualizing cold spots: TRPM8-expressing sensory neurons and their projections. J Neurosci 28:566–575. 10.1523/JNEUROSCI.3976-07.2008 18199758 PMC6670358

[B20] Dhaka A, Murray AN, Mathur J, Earley TJ, Petrus MJ, Patapoutian A (2007) TRPM8 is required for cold sensation in mice. Neuron 54:371–378. 10.1016/j.neuron.2007.02.024 17481391

[B21] Dobler T, Springauf A, Tovornik S, Weber M, Schmitt A, Sedlmeier R, Wischmeyer E, Döring F (2007) TRESK two-pore-domain K+ channels constitute a significant component of background potassium currents in murine dorsal root ganglion neurones. J Physiol 585:867–879. 10.1113/jphysiol.2007.145649 17962323 PMC2375503

[B22] Edvinsson L, Goadsby PJ (2019) Discovery of CGRP in relation to migraine. Cephalalgia 39:331–332. 10.1177/0333102418779544 30827155

[B23] Elliott MB, Oshinsky ML, Amenta PS, Awe OO, Jallo JI (2012) Nociceptive neuropeptide increases and periorbital allodynia in a model of traumatic brain injury. Headache 52:966–984. 10.1111/j.1526-4610.2012.02160.x 22568499 PMC4105160

[B24] Enyedi P, Czirják G (2015) Properties, regulation, pharmacology, and functions of the K(2)p channel, TRESK. Pflugers Archiv 467:945–958. 10.1007/s00424-014-1634-8 25366493

[B25] Fang X, Djouhri L, McMullan S, Berry C, Waxman SG, Okuse K, Lawson SN (2006) Intense isolectin-B4 binding in rat dorsal root ganglion neurons distinguishes C-fiber nociceptors with broad action potentials and high Nav1.9 expression. J Neurosci 26:7281–7292. 10.1523/JNEUROSCI.1072-06.2006 16822986 PMC6673936

[B26] Flegel C, Schöbel N, Altmüller J, Becker C, Tannapfel A, Hatt H, Gisselmann G (2015) RNA-Seq Analysis of Human Trigeminal and Dorsal Root Ganglia with a Focus on Chemoreceptors. PloS One 10:e0128951. 10.1371/journal.pone.0128951 26070209 PMC4466559

[B27] Franceschini A, Vilotti S, Ferrari MD, van den Maagdenberg AM, Nistri A, Fabbretti E (2013) TNFα levels and macrophages expression reflect an inflammatory potential of trigeminal ganglia in a mouse model of familial hemiplegic migraine. PloS One 8:e52394 10.1371/journal.pone.0052394 23326332 PMC3543418

[B28] Golden JP, Hoshi M, Nassar MA, Enomoto H, Wood JN, Milbrandt J, Gereau RWt, Johnson EM Jr, Jain S (2010) RET signaling is required for survival and normal function of nonpeptidergic nociceptors. J Neurosci 30:3983–3994. 10.1523/JNEUROSCI.5930-09.2010 20237269 PMC2850282

[B29] Goldstein ME, House SB, Gainer H (1991) NF-L and peripherin immunoreactivities define distinct classes of rat sensory ganglion cells. J Neurosci Res 30:92–104. 10.1002/jnr.490300111 1795410

[B30] Gracely RH, Lynch SA, Bennett GJ (1992) Painful neuropathy: altered central processing maintained dynamically by peripheral input. Pain 51:175–194. 1484715 10.1016/0304-3959(92)90259-E

[B31] Guo Z, Cao YQ (2014) Over-expression of TRESK K(+) channels reduces the excitability of trigeminal ganglion nociceptors. PloS One 9:e87029. 10.1371/journal.pone.0087029 24466320 PMC3900698

[B32] Guo Z, Liu P, Ren F, Cao YQ (2014) Non-migraine associated TRESK K+ channel variant C110R does not increase the excitability of trigeminal ganglion neurons. J Neurophysiol 112:568–579. 10.1152/jn.00267.2014 24805079 PMC4122697

[B33] Hargreaves K, Dubner R, Brown F, Flores C, Joris J (1988) A new and sensitive method for measuring thermal nociception in cutaneous hyperalgesia. Pain 32:77–88. 3340425 10.1016/0304-3959(88)90026-7

[B34] Haroutounian S, Nikolajsen L, Bendtsen TF, Finnerup NB, Kristensen AD, Hasselstrøm JB, Jensen TS (2014) Primary afferent input critical for maintaining spontaneous pain in peripheral neuropathy. Pain 155:1272–1279. 10.1016/j.pain.2014.03.022 24704366

[B35] Harper AA, Lawson SN (1985a) Conduction velocity is related to morphological cell type in rat dorsal root ganglion neurones. J Physiol 359:31–46. 10.1113/jphysiol.1985.sp015573 3999040 PMC1193363

[B36] Harper AA, Lawson SN (1985b) Electrical properties of rat dorsal root ganglion neurones with different peripheral nerve conduction velocities. J Physiol 359:47–63. 10.1113/jphysiol.1985.sp015574 2987489 PMC1193364

[B37] Huang CC, Yang W, Guo C, Jiang H, Li F, Xiao M, Davidson S, Yu G, Duan B, Huang T, Huang AJW, Liu Q (2018) Anatomical and functional dichotomy of ocular itch and pain. Nat Med 24:1268–1276. 10.1038/s41591-018-0083-x 29988128 PMC6093777

[B38] Huang D, Li SY, Dhaka A, Story GM, Cao YQ (2012) Expression of the transient receptor potential channels TRPV1, TRPA1 and TRPM8 in mouse trigeminal primary afferent neurons innervating the dura. Mol Pain 8:66 10.1186/1744-8069-8-66 22971321 PMC3489865

[B39] Huang D, Ren L, Qiu CS, Liu P, Peterson J, Yanagawa Y, Cao YQ (2016) Characterization of a mouse model of headache. Pain 157:1744–1760. 10.1097/j.pain.0000000000000578 27058678 PMC4960827

[B40] Kang D, Kim D (2006) TREK-2 (K2P10.1) and TRESK (K2P18.1) are major background K+ channels in dorsal root ganglion neurons. Am J Physiol 291:C138–C146. 10.1152/ajpcell.00629.2005 16495368

[B41] Kang D, Mariash E, Kim D (2004) Functional expression of TRESK-2, a new member of the tandem-pore K+ channel family. J Biol Chem 279:28063–28070. 10.1074/jbc.M402940200 15123670

[B42] Knowlton WM, Palkar R, Lippoldt EK, McCoy DD, Baluch F, Chen J, McKemy DD (2013) A sensory-labeled line for cold: TRPM8-expressing sensory neurons define the cellular basis for cold, cold pain, and cooling-mediated analgesia. J Neurosci 33:2837–2848. 10.1523/JNEUROSCI.1943-12.2013 23407943 PMC3711390

[B43] Kollert S, Dombert B, Döring F, Wischmeyer E (2015) Activation of TRESK channels by the inflammatory mediator lysophosphatidic acid balances nociceptive signalling. Sci Rep 5:12548. 10.1038/srep12548 26224542 PMC4519772

[B44] Lafrenière RG, Cader MZ, Poulin JF, Andres-Enguix I, Simoneau M, Gupta N, Boisvert K, Lafrenière F, McLaughlan S, Dubé MP, Marcinkiewicz MM, Ramagopalan S, Ansorge O, Brais B, Sequeiros J, Pereira-Monteiro JM, Griffiths LR, Tucker SJ, Ebers G, Rouleau GA (2010) A dominant-negative mutation in the TRESK potassium channel is linked to familial migraine with aura. Nat Med 16:1157–1160. 10.1038/nm.2216 20871611

[B45] LaPaglia DM, Sapio MR, Burbelo PD, Thierry-Mieg J, Thierry-Mieg D, Raithel SJ, Ramsden CE, Iadarola MJ, Mannes AJ (2018) RNA-Seq investigations of human post-mortem trigeminal ganglia. Cephalalgia 38:912–932. 10.1177/0333102417720216 28699403 PMC6326384

[B46] Lawson SN, Crepps BA, Perl ER (1997) Relationship of substance P to afferent characteristics of dorsal root ganglion neurones in guinea-pig. J Physiol 505: 177–191. 10.1111/j.1469-7793.1997.00177.x 9409481 PMC1160103

[B47] Le Pichon CE, Chesler AT (2014) The functional and anatomical dissection of somatosensory subpopulations using mouse genetics. Front Neuroanat 8:21. 10.3389/fnana.2014.00021 24795573 PMC4001001

[B48] Lennertz RC, Tsunozaki M, Bautista DM, Stucky CL (2010) Physiological basis of tingling paresthesia evoked by hydroxy-alpha-sanshool. J Neurosci 30:4353–4361. 10.1523/JNEUROSCI.4666-09.2010 20335471 PMC2852189

[B49] Liu P, Xiao Z, Ren F, Guo Z, Chen Z, Zhao H, Cao YQ (2013) Functional analysis of a migraine-associated TRESK K+ channel mutation. J Neurosci 33:12810–12824. 10.1523/JNEUROSCI.1237-13.2013 23904616 PMC3728689

[B50] Lopes DM, Denk F, McMahon SB (2017) The molecular fingerprint of dorsal root and trigeminal ganglion neurons. Front Mol Neurosci 10:304. 10.3389/fnmol.2017.00304 29018326 PMC5623188

[B51] Madrid R, de la Pena E, Donovan-Rodriguez T, Belmonte C, Viana F (2009) Variable threshold of trigeminal cold-thermosensitive neurons is determined by a balance between TRPM8 and Kv1 potassium channels. J Neurosci 29:3120–3131. 10.1523/JNEUROSCI.4778-08.2009 19279249 PMC6666436

[B52] Marsh B, Acosta C, Djouhri L, Lawson SN (2012) Leak K(+) channel mRNAs in dorsal root ganglia: relation to inflammation and spontaneous pain behaviour. Mol Cell Neurosci 49:375–386. 10.1016/j.mcn.2012.01.002 22273507 PMC3334831

[B53] Martinez-Espinosa PL, Wu J, Yang C, Gonzalez-Perez V, Zhou H, Liang H, Xia XM, Lingle CJ (2015) Knockout of Slo2.2 enhances itch, abolishes KNa current, and increases action potential firing frequency in DRG neurons. eLife 4:e10013. 26559620 10.7554/eLife.10013 PMC4641468

[B54] McCoy ES, Taylor-Blake B, Street SE, Pribisko AL, Zheng J, Zylka MJ (2013) Peptidergic CGRPα primary sensory neurons encode heat and itch and tonically suppress sensitivity to cold. Neuron 78:138–151. 10.1016/j.neuron.2013.01.030 23523592 PMC3628403

[B55] Morenilla-Palao C, Luis E, Fernández-Peña C, Quintero E, Weaver JL, Bayliss DA, Viana F (2014) Ion channel profile of TRPM8 cold receptors reveals a role of TASK-3 potassium channels in thermosensation. Cell Rep 8:1571–1582. 10.1016/j.celrep.2014.08.003 25199828 PMC5724366

[B56] Noël J, Zimmermann K, Busserolles J, Deval E, Alloui A, Diochot S, Guy N, Borsotto M, Reeh P, Eschalier A, Lazdunski M (2009) The mechano-activated K+ channels TRAAK and TREK-1 control both warm and cold perception. EMBO J 28:1308–1318. 10.1038/emboj.2009.57 19279663 PMC2683043

[B57] Odem MA, Bavencoffe AG, Cassidy RM, Lopez ER, Tian J, Dessauer CW, Walters ET (2018) Isolated nociceptors reveal multiple specializations for generating irregular ongoing activity associated with ongoing pain. Pain 159:2347–2362. 10.1097/j.pain.0000000000001341 30015712 PMC6193853

[B58] Pereira V, Busserolles J, Christin M, Devilliers M, Poupon L, Legha W, Alloui A, Aissouni Y, Bourinet E, Lesage F, Eschalier A, Lazdunski M, Noël J (2014) Role of the TREK2 potassium channel in cold and warm thermosensation and in pain perception. Pain 155:2534–2544. 10.1016/j.pain.2014.09.013 25239074

[B59] Perry MJ, Lawson SN, Robertson J (1991) Neurofilament immunoreactivity in populations of rat primary afferent neurons: a quantitative study of phosphorylated and non-phosphorylated subunits. J Neurocytol 20:746–758. 1960537 10.1007/BF01187848

[B60] Pogorzala LA, Mishra SK, Hoon MA (2013) The cellular code for mammalian thermosensation. J Neurosci 33:5533–5541. 10.1523/JNEUROSCI.5788-12.2013 23536068 PMC3642850

[B61] Price TJ, Flores CM (2007) Critical evaluation of the colocalization between calcitonin gene-related peptide, substance P, transient receptor potential vanilloid subfamily type 1 immunoreactivities, and isolectin B4 binding in primary afferent neurons of the rat and mouse. J Pain 8:263–272. 10.1016/j.jpain.2006.09.005 17113352 PMC1899162

[B62] Rainero I, Rubino E, Gallone S, Zavarise P, Carli D, Boschi S, Fenoglio P, Savi L, Gentile S, Benna P, Pinessi L, Dalla Volta G (2014) KCNK18 (TRESK) genetic variants in Italian patients with migraine. Headache 54:1515–1522. 10.1111/head.12439 25324165

[B63] Ratté S, Zhu Y, Lee KY, Prescott SA (2014) Criticality and degeneracy in injury-induced changes in primary afferent excitability and the implications for neuropathic pain. eLife 3:e02370. 10.7554/eLife.02370 24692450 PMC3970756

[B64] Rossi A, Kontarakis Z, Gerri C, Nolte H, Hölper S, Krüger M, Stainier DY (2015) Genetic compensation induced by deleterious mutations but not gene knockdowns. Nature 524:230–233. 10.1038/nature14580 26168398

[B65] Royal P, Andres-Bilbe A, ÁvalosPrado P, Verkest C, Wdziekonski B, Schaub S, Baron A, Lesage F, Gasull X, Levitz J, Sandoz G (2019) Migraine-associated TRESK mutations increase neuronal excitability through alternative translation initiation and inhibition of TREK. Neuron 101:232–245.e6. 10.1016/j.neuron.2018.11.039 30573346

[B66] Sano Y, Inamura K, Miyake A, Mochizuki S, Kitada C, Yokoi H, Nozawa K, Okada H, Matsushime H, Furuichi K (2003) A novel two-pore domain K+ channel, TRESK, is localized in the spinal cord. J Biol Chem 278:27406–27412. 10.1074/jbc.M206810200 12754259

[B67] Scherrer G, Imamachi N, Cao YQ, Contet C, Mennicken F, O'Donnell D, Kieffer BL, Basbaum AI (2009) Dissociation of the opioid receptor mechanisms that control mechanical and heat pain. Cell 137:1148–1159. 10.1016/j.cell.2009.04.019 19524516 PMC3683597

[B68] Seal RP, Wang X, Guan Y, Raja SN, Woodbury CJ, Basbaum AI, Edwards RH (2009) Injury-induced mechanical hypersensitivity requires C-low threshold mechanoreceptors. Nature 462:651–655. 10.1038/nature08505 19915548 PMC2810205

[B69] Shimada SG, LaMotte RH (2008) Behavioral differentiation between itch and pain in mouse. Pain 139:681–687. 10.1016/j.pain.2008.08.002 18789837 PMC2723192

[B70] Snider WD, McMahon SB (1998) Tackling pain at the source: new ideas about nociceptors. Neuron 20:629–632. 9581756 10.1016/s0896-6273(00)81003-x

[B71] Stucky CL, Lewin GR (1999) Isolectin B(4)-positive and -negative nociceptors are functionally distinct. J Neurosci 19:6497–6505. 10.1523/JNEUROSCI.19-15-06497.1999 10414978 PMC6782829

[B72] Tao J, Liu P, Xiao Z, Zhao H, Gerber BR, Cao YQ (2012) Effects of familial hemiplegic migraine type 1 mutation T666M on voltage-gated calcium channel activities in trigeminal ganglion neurons. J Neurophysiol 107:1666–1680. 10.1152/jn.00551.2011 22190617 PMC3311679

[B73] Trevisan G, Benemei S, Materazzi S, De Logu F, De Siena G, Fusi C, Fortes Rossato M, Coppi E, Marone IM, Ferreira J, Geppetti P, Nassini R (2016) TRPA1 mediates trigeminal neuropathic pain in mice downstream of monocytes/macrophages and oxidative stress. Brain 139:1361–1377. 10.1093/brain/aww038 26984186

[B74] Tulleuda A, Cokic B, Callejo G, Saiani B, Serra J, Gasull X (2011) TRESK channel contribution to nociceptive sensory neurons excitability: modulation by nerve injury. Mol Pain 7:30. 10.1186/1744-8069-7-30 21527011 PMC3095542

[B75] Usoskin D, Furlan A, Islam S, Abdo H, Lönnerberg P, Lou D, Hjerling-Leffler J, Haeggström J, Kharchenko O, Kharchenko PV, Linnarsson S, Ernfors P (2015) Unbiased classification of sensory neuron types by large-scale single-cell RNA sequencing. Nat Neurosci 18:145–153. 10.1038/nn.3881 25420068

[B76] Valenzuela DM, Murphy AJ, Frendewey D, Gale NW, Economides AN, Auerbach W, Poueymirou WT, Adams NC, Rojas J, Yasenchak J, Chernomorsky R, Boucher M, Elsasser AL, Esau L, Zheng J, Griffiths JA, Wang X, Su H, Xue Y, Dominguez MG, et al. (2003) High-throughput engineering of the mouse genome coupled with high-resolution expression analysis. Nat Biotechnol 21:652–659. 10.1038/nbt822 12730667

[B77] Vetter I, Hein A, Sattler S, Hessler S, Touska F, Bressan E, Parra A, Hager U, Leffler A, Boukalova S, Nissen M, Lewis RJ, Belmonte C, Alzheimer C, Huth T, Vlachova V, Reeh PW, Zimmermann K (2013) Amplified cold transduction in native nociceptors by M-channel inhibition. J Neurosci 33:16627–16641. 10.1523/JNEUROSCI.1473-13.2013 24133266 PMC6618521

[B78] Viana F, de la Peña E, Belmonte C (2002) Specificity of cold thermotransduction is determined by differential ionic channel expression. Nat Neurosci 5:254–260. 10.1038/nn809 11836533

[B79] Yoo S, Liu J, Sabbadini M, Au P, Xie GX, Yost CS (2009) Regional expression of the anesthetic-activated potassium channel TRESK in the rat nervous system. Neurosci Lett 465:79–84. 10.1016/j.neulet.2009.08.062 19716403 PMC2778220

[B80] Zhang J, Cavanaugh DJ, Nemenov MI, Basbaum AI (2013) The modality-specific contribution of peptidergic and non-peptidergic nociceptors is manifest at the level of dorsal horn nociresponsive neurons. J Physiol 591:1097–1110. 10.1113/jphysiol.2012.242115 23266932 PMC3591717

[B81] Zhang XL, Mok LP, Katz EJ, Gold MS (2010) BK_Ca_ currents are enriched in a subpopulation of adult rat cutaneous nociceptive dorsal root ganglion neurons. Eur J Neurosci 31:450–462. 10.1111/j.1460-9568.2009.07060.x 20105244 PMC2843514

[B82] Zhou J, Yang CX, Zhong JY, Wang HB (2013) Intrathecal TRESK gene recombinant adenovirus attenuates spared nerve injury-induced neuropathic pain in rats. Neuroreport 24:131–136. 10.1097/WNR.0b013e32835d8431 23370493

[B83] Zhou J, Lin W, Chen H, Fan Y, Yang C (2016) TRESK contributes to pain threshold changes by mediating apoptosis via MAPK pathway in the spinal cord. Neuroscience 339:622–633. 10.1016/j.neuroscience.2016.10.039 27789381

[B84] Zhou J, Yao SL, Yang CX, Zhong JY, Wang HB, Zhang Y (2012) TRESK gene recombinant adenovirus vector inhibits capsaicin-mediated substance P release from cultured rat dorsal root ganglion neurons. Mol Med Rep 5:1049–1052. 10.3892/mmr.2012.778 22307830 PMC3493032

